# Semiconducting Covalent Organic Frameworks Based on Spin‐Delocalized Trioxotriangulene Neutral Radicals

**DOI:** 10.1002/anie.6104022

**Published:** 2026-08-10

**Authors:** Paula Escamilla, Sara Trigo‐Pérez, Rafael Ramos, Ricardo Ortiz, Manuel Vilas‐Varela, Diego Peña, Manuel Melle‐Franco, Francisco Rivadulla, Manuel Souto

**Affiliations:** ^1^ Centro Singular De Investigación en Química Biolóxica e Materiais Moleculares (CiQUS), Departamento de Química‐Física Universidade De Santiago De Compostela Santiago de Compostela Spain; ^2^ Department of Chemistry, CICECO‐Aveiro Institute of Materials University of Aveiro Aveiro Portugal; ^3^ Oportunius, Galician Innovation Agency (GAIN) Santiago de Compostela Spain

**Keywords:** covalent organic framework, electrical conductivity, neutral radical, spin‐delocalized radical, trioxotriangulene

## Abstract

Electrically conductive two‐dimensional covalent organic frameworks (2D COFs) have emerged as a versatile class of crystalline porous polymers with promising applications in electronics and energy storage. However, high electrical conductivity generally relies on post‐synthetic doping to generate charge carriers, which can compromise crystallinity, porosity, and structural homogeneity. Persistent neutral radical conductors provide an attractive alternative, as their unpaired electrons can generate free charge carriers without the need for counterions. Nevertheless, the incorporation of highly spin‐delocalized π‐radicals into COFs remains largely unexplored. Herein, we report the design and synthesis of imine‐linked 2D COFs incorporating spin‐delocalized trioxotriangulene (TOT) neutral radicals through complementary synthetic approaches. Direct use of a TOT radical derivative bearing amino groups affords a highly crystalline framework (TOT‐COF‐H) that exhibits semiconducting behavior (*σ*_RT_ = 1.2 × 10^−4^ S cm^−1^), a reduced band gap (*E*_g_ = 1.09 eV), and a low activation energy (0.24 eV). This work demonstrates a viable strategy for integrating spin‐delocalized neutral radical building blocks into COFs, enabling the development of intrinsically conductive, porous, and crystalline organic frameworks without the need for extrinsic doping.

## Graphical abstract

Imine‐linked 2D covalent organic frameworks incorporating delocalized trioxotriangulene (TOT) neutral radicals were synthesized through complementary routes. Direct incorporation of an amine‐functionalized TOT radical yields a highly crystalline, porous framework (TOT‐COF‐H) with intrinsic semiconducting behavior (*σ*_RT_ = 1.2 × 10^−4^ S cm^−1^) and a narrow band gap (*E*_g_ = 1.09 eV), eliminating the need for extrinsic doping.

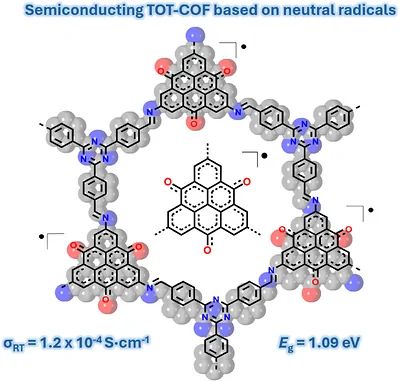

## 1 Introduction

Covalent organic frameworks (COFs) are crystalline porous polymers constructed from organic building blocks connected by strong covalent bonds, enabling a wide range of applications [1, 2, 3]. In particular, electrically conductive two‐dimensional (2D) COFs have garnered considerable attention due to their tunable electronic structures and potential in electronics and energy‐related applications [4, 5, 6, 7, 8]. However, in most cases, chemical doping (e.g., I_2_, TCNQ) is required to increase the concentration of free charge carriers, which can result in decreased porosity due to the presence of counterions, reduced crystallinity, or inhomogeneous doping [5, 9, 10, 11, 12, 13]. Therefore, new strategies for introducing charge carriers into the framework beyond post‐synthetic doping are required to preserve the crystallinity and porosity of conductive COFs.

Persistent neutral organic radicals have long been explored as single‐component conductors because the unpaired electron can give rise to a half‐filled band with free charge carriers, eliminating the need for counterions [14, 15, 16, 17, 18, 19]. While most organic radicals behave as Mott insulators, certain highly spin‐delocalized planar radicals can exhibit high electrical conductivities [20, 21, 22]. In this context, Morita and co‐workers reported the synthesis of various 4,8,12‐trioxotriangulene (TOT) neutral radical derivatives (Scheme 1), which show high stability under ambient conditions owing to spin delocalization over the entire π‐electronic system [23, 24, 25, 26]. These neutral radicals can form one‐dimensional π‐stacking columns with strong intermolecular orbital overlap, achieving room‐temperature conductivities of up to ∼10^−3^ S cm^−1^ [24]. In addition, open‐shell TOT derivatives exhibit multiple redox processes, making them promising candidates for high‐capacity organic electrode materials in metal‐ion batteries [23].

**SCHEME 1:**
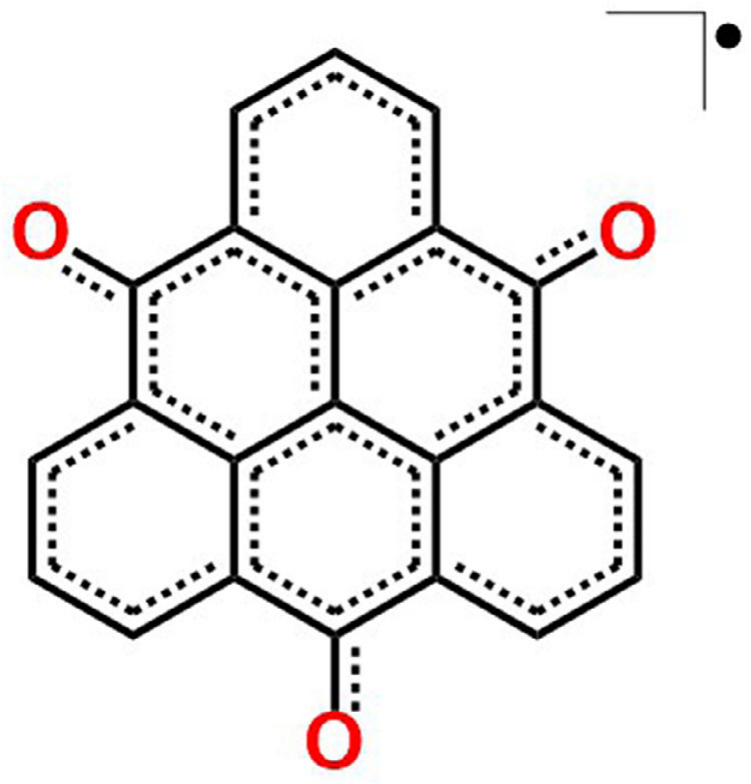
Molecular structure of trioxotriangulene (TOT) neutral radical.

The unique combination of optical, electronic, and magnetic properties exhibited by persistent organic radicals renders them attractive building blocks for the development of functional COFs [4, 27]. In this context, only a few COFs based on spin‐localized radicals such as 2,2,6,6 tetramethylpiperidine‐1‐oxyl (TEMPO) [28, 29, 30] or perchlorotriphenylmethyl [31, 32] moieties have been reported. However, the incorporation of highly delocalized, planar polycyclic π‐radicals into COFs remains unexplored, despite their potential to increase their electrical conductivity without the need for extrinsic doping. In this regard, although some theoretical studies predict promising electrical and magnetic properties of TOT radical frameworks [33, 34], TOT radical building blocks have not yet been integrated into COFs.

Herein, we report a new 2D COF based on highly delocalized TOT neutral radicals that can be obtained through two different synthetic approaches (Scheme 2). The imine‐linked **TOT‐COF** was prepared either from a TOT salt functionalized with benzophenone imine groups or from a deprotected TOT radical bearing free amino groups (Scheme 2). The latter approach afforded a COF with higher crystallinity (**TOT‐COF‐H**) than that obtained from the benzophenone imine precursor (**TOT‐COF‐L**). **TOT‐COF‐H** exhibits enhanced electrical conductivity (*σ*_RT_ = 1.2 x 10^−4^ S cm^−1^), a reduced band gap (*E*_g_ = 1.09 eV), and a low activation energy (241 meV). These results demonstrate the potential of incorporating delocalized π‐radicals into COFs to design semiconducting neutral frameworks while preserving high crystallinity, porosity, and thermal stability.

**SCHEME 2:**
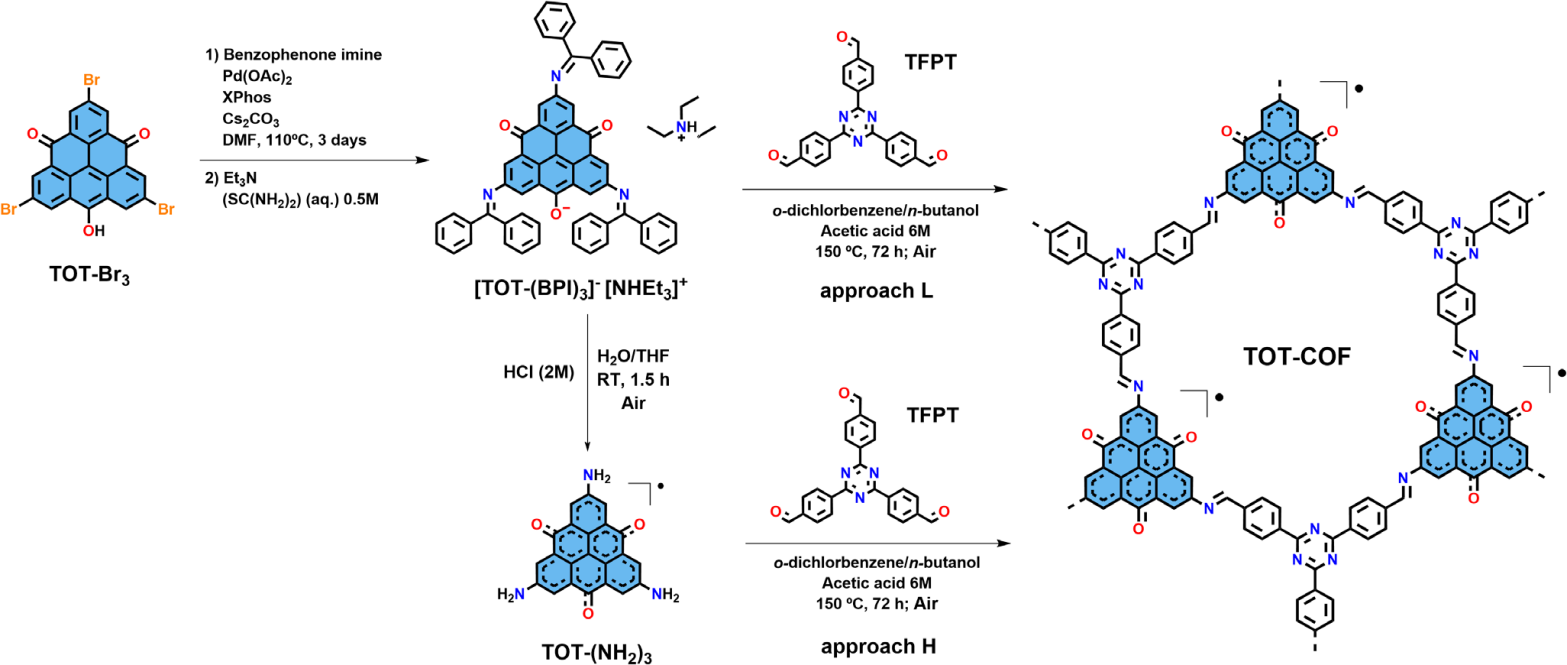
Synthesis of **TOT‐COF**.

## 2 Results and Discussion

### 2.1 Synthesis

To enable the incorporation of TOT radicals into the imine‐linked COF, benzophenone imine (BPI)‐ and triamino‐functionalized TOT derivatives were synthesized (Scheme 2). First, **TOT‐(BPI)_3_** was prepared via a Buchwald–Hartwig cross‐coupling reaction between **TOT‐Br_3_** [23] and benzophenone imine. Subsequently, **TOT‐(BPI)_3_** was treated with triethylamine to afford the corresponding triethylammonium salt [**TOT‐(BPI)_3_]^−^[NHEt_3_]^+^**, which was fully characterized (Figures S1 and S2). Hydrolysis of **[TOT‐(BPI)_3_]^−^[NHEt_3_]^+^** yielded the amino‐functionalized **TOT‐(NH_2_)_3_** derivative. During hydrolysis, the TOT radical species were generated by oxidation in air, as confirmed by electron spin resonance (ESR) spectroscopy (see discussion below).

Two different synthetic routes were employed to obtain the **TOT‐COF** (Scheme 2). In both cases, the **TOT‐COF** was obtained under solvothermal conditions through the condensation reaction of 2,4,6‐tris(4‐formylphenyl)‐1,3,5‐triazine (**TFPT**) and **TOT‐(NH_2_)_3_** or **[TOT‐(BPI)_3_]^−^[NHEt_3_]^+^** in a mixture of *o*‐dichlorobenzene, *n*‐butanol, and 6 M aqueous acetic acid (Scheme 2). The reactions were carried out in sealed Pyrex tubes under air at 150°C for 72 h, with molecular oxygen serving as the oxidant during the formation of the radical framework. The resulting dark blue powders were sequentially washed with DMF, THF, dichloromethane, and acetone to remove unreacted reagents, and then dried under vacuum at 100°C for 12 h.

### 2.2 Physicochemical Characterization

Powder X‐ray diffraction (PXRD) measurements were carried out to evaluate the crystallinity of the synthesized **TOT‐COF** (Figure 1a). PXRD patterns of the materials obtained through the two synthetic approaches exhibit similar diffraction features, with an intense reflection at 2*θ*  =  4.4° and weaker peaks at 7.7°, 8.9°, 11.7°, 16.1°, and 26.3°, corresponding to the (100), (110), (200), (210), (220), and (001) planes, respectively. The primary difference between the two materials (Scheme 2) is their degree of crystallinity: **TOT‐COF‐H**, synthesized from **TOT‐(NH_2_)_3_**, exhibits significantly higher crystallinity than **TOT‐COF‐L**, prepared from **[TOT‐(BPI)_3_]^−^[NHEt_3_]^+^**. Although substituted benzophenone imines typically result in crystalline COFs [35], the lower crystallinity observed for **TOT‐COF‐L** is likely associated with differences in the crystallization process, which may be influenced by factors such as precursor solubility and reaction kinetics. Simulated PXRD patterns generated from a parallel‐displaced AA‐stacking (pdAA) model are in good agreement with the experimental data (Figure 1a). Pawley refinement of the unit cell parameters was performed to closely match the experimental PXRD patterns, providing a reasonable indexing of the observed diffraction features between the calculated and observed patterns (Figure S3, Table S1).

**FIGURE 1:**
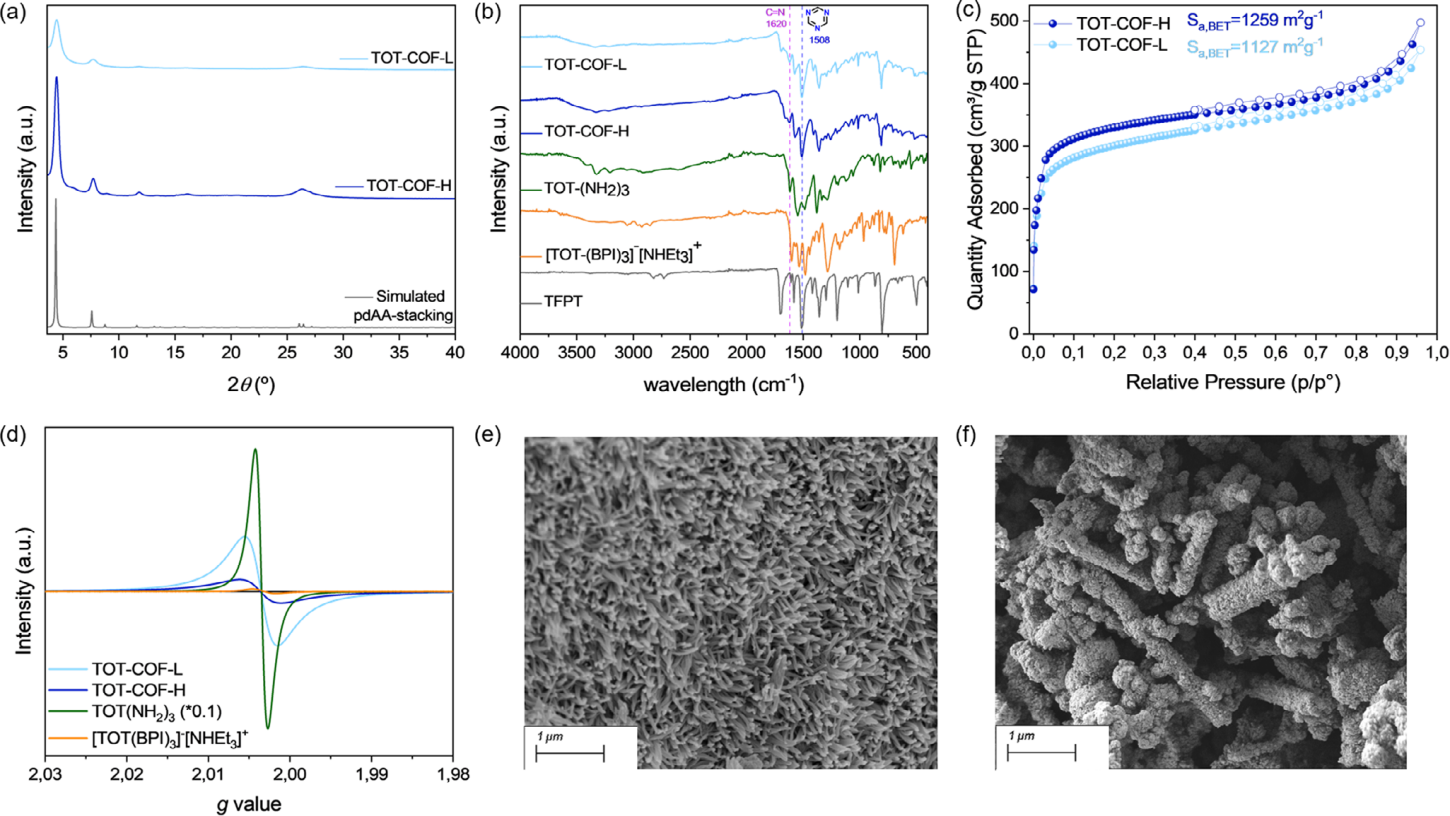
Characterization of **TOT‐COF‐H** and **TOT‐COF‐L**. (a) Experimental (dark and light blue) and simulated (gray) pdAA stacking PXRD patterns. (b) FT‐IR spectra. (c) N_2_ adsorption‐desorption isotherms at 77 K. (d) ESR spectra at room temperature. The signal of **TOT‐(NH_2_)_3_** was multiplied by a factor of 0.1 for comparison. (e) SEM image of **TOT‐COF‐H**. (f) SEM image of **TOT‐COF‐L**.

Fourier transform infrared (FT‐IR) spectra of **TOT‐COF‐H** and **TOT‐COF‐L** were recorded and compared with those of their corresponding precursors (Figure 1b). The disappearance of the characteristic −CHO (1685 cm^−^^1^), −NH_2_ (3500–3300 cm^−^^1^), and aliphatic −CH (2850–2950 cm^−^^1^) stretching bands, concurrent with the appearance of a new band at 1620 cm^−^^1^ assigned to the C═N stretching, clearly confirms the successful formation of imine linkages within the **TOT‐COF** frameworks. Solid‐state cross‐polarization magic‐angle‐spinning (CP‐MAS) ^13^C NMR spectroscopy further confirmed the chemical structure of the COFs (Figure S4). The NMR spectrum shows a characteristic resonance in the ∼155–162 ppm region, assigned to the imine (C═N) carbon, validating successful Schiff base condensation. The NMR signals observed at ∼175–185 ppm are attributed to the carbonyl carbons of the TOT radical core, resulting in an upfield shift relative to typical carbonyl chemical shifts, whereas the signal at ∼190 ppm can be related to residual non‐radical carbonyl species, indicating incomplete radical formation, in agreement with quantitative ESR results (see below). Resonances in the 100–150 ppm range correspond to the aromatic carbons of the linker units.

The permanent porosity of **TOT‐COF‐H** and **TOT‐COF‐L** was evaluated by nitrogen adsorption–desorption isotherms at 77 K (Figure 1c). The Brunauer–Emmett–Teller (BET) specific surface areas were calculated using BETSI analysis [36], yielding values of 1259 and 1127 m^2^ g^−^^1^ for **TOT‐COF‐H** and **TOT‐COF‐L**, respectively (Figures S5 and S6), in good agreement with the results computed from the model, which yielded a calculated BET surface area of 1453 m^2^ g^−^^1^. Despite its lower crystallinity, **TOT‐COF‐L** retains a comparable BET surface area, indicating that the reduction in long‐range order is primarily associated with smaller crystalline domains and/or stacking disorder rather than collapse of the porous framework. The pore size distribution (PSD) studies show maxima at approximately 1.2 and 1.6 nm for both COFs (Figure S7), in agreement with a reported isostructural COF [37]. Although the structural model predicts a single pore with a diameter of ∼1.4 nm, the adsorption‐derived PSD indicates a distribution of pore environments around this value. The presence of these two maxima can be attributed to local structural heterogeneity, such as framework defects. Thermogravimetric analysis (TGA) revealed that both materials exhibit remarkable thermal stability, with no significant weight loss observed up to 360°C (Figure S8), which is consistent with the high stability typically reported for TOT radical molecules [23].

Scanning electron microscopy (SEM) images reveal distinct morphological differences between the synthesized COFs. **TOT‐COF‐H** (Figure 1e) exhibits densely packed, well‐ordered needle‐like crystals, whereas **TOT‐COF‐L** (Figure 1f) forms micrometer‐sized irregular aggregates. These differences in morphology and crystallinity likely arise from distinct nucleation processes during framework formation. This needle‐like morphology has previously been observed in similar trioxaazatriangulene‐based COFs and is associated with a templated growth of COFs on the crystal surface of the poorly soluble precursors [38, 39]. High‐resolution transmission electron microscopy (HRTEM) images of **TOT‐COF‐H** and **TOT‐COF‐L** (Figure S9) display well‐defined periodic lattice fringes extending over large domains, providing direct real‐space evidence of the high crystallinity of these framework materials.

### 2.3 Magnetic Properties of TOT‐COFs

ESR spectroscopy of the precursors, **TOT‐COF‐H** and **TOT‐COF‐L** was performed to quantify the density of radical species within the framework (Figures 1d and S10, Table S2). The **[TOT‐(BPI)_3_]^−^[NHEt_3_]^+^** salt exhibited a very weak signal corresponding to 5.66 × 10^−^^3^ spins per molecule, which is consistent with the absence of radical units. In contrast, the related **TOT‐(NH_2_)_3_** radical derivative displayed an intense ESR signal corresponding to 0.48 spins per molecule, indicating that approximately half of the radical sites are ESR‐active under the measurement conditions, likely due to partial spin quenching arising from antiferromagnetic interactions, as observed for related TOT radicals [24, 25], or incomplete radical formation. This remarkable increase in intensity further confirmed the formation of TOT radical species upon oxidation by air.

The successful incorporation of TOT radicals within the COF was further confirmed by ESR. ESR quantification revealed 0.39 and 0.68 spins per unit cell for **TOT‐COF‐H** and **TOT‐COF‐L** (Table S2), respectively. Given that each unit cell contains two TOT units, these values suggest that approximately 20% and 34% of the radical sites in **TOT‐COF‐H** and **TOT‐COF‐L**, respectively, are ESR active. It is important to note that these fractional spin values should not be interpreted as exact radical yields, but rather as the experimentally accessible fraction of ESR‐active spins, which can be influenced by incomplete radical formation, partial deactivation of radical sites, and antiferromagnetic interactions. All samples exhibited a *g* = 2.003, close to the free‐electron value and characteristic of carbon‐centered organic radicals [39]. Temperature‐dependent magnetic susceptibility measurements revealed that **TOT‐COF‐H** and **TOT‐COF‐L** exhibit predominantly paramagnetic behavior. A gradual decrease of *χ*_m_ *T* upon cooling was observed, which is indicative of weak antiferromagnetic interactions between the radical units (Curie constant of *C* = 0.21 cm^3^ K mol^−1^ for both TOT‐COFs) (Figure S11), in good agreement with theoretical calculations (see below). The *χ*_m_ *T* value of **TOT‐COF‐H** remains nearly constant over the entire temperature range, indicating that the radical spins behave largely as weakly interacting paramagnetic centers. In contrast, **TOT‐COF‐L** exhibits a more pronounced temperature dependence of *χ*_m_ *T*, suggesting stronger antiferromagnetic interactions between radical sites, probably related to its higher radical concentration. Curie–Weiss analysis afforded Weiss temperatures of *θ* = −2.22 K for **TOT‐COF‐H** and *θ* = −7.88 K for **TOT‐COF‐L**, demonstrating weak antiferromagnetic interactions in both materials, which are significantly stronger in **TOT‐COF‐L**. The reduced ESR intensity and sub‐ideal Curie constant are likely attributed to partial spin quenching [24, 25], defects, and/or incomplete radical formation. We investigated whether the ESR signal could be enhanced by post‐synthetic deprotonation and oxidation while preserving the crystallinity of the framework. Specifically, **TOT‐COF‐H** was treated with tetrabutylammonium hydroxide in THF, followed by oxidation with *p*‐chloranil. However, this treatment did not result in a significant increase in the ESR signal, while the PXRD pattern revealed a slight loss of crystallinity (Figure S12).

### 2.4 Electronic Properties of TOT‐COFs

Cyclic voltammetry (CV) and differential pulse voltammetry (DPV) of the triethylammonium salt of **TOT‐(BPI)_3_** and **TOT‐COF‐H** were performed to investigate their electrochemical properties (Figure S13). CV of **[TOT‐(BPI)_3_]^−^[NHEt_3_]^+^** exhibits a well‐defined, reversible one‐electron oxidation process (around –0.2 V vs. Fc/Fc^+^), corresponding to the formation of the TOT radical species and consistent with the behavior reported for other TOT anion salts [23]. In contrast, the reduction processes at more negative potentials are assigned to successive one‐electron reductions of the TOT anion, which are only quasi‐reversible or irreversible, likely due to limited solubility and decreased stability of the highly reduced species, as observed for related TOT systems [23]. Otherwise, solid‐state CV and DPV of **TOT‐COF‐H** show less intense and less well‐defined redox peaks at similar potentials, suggesting that the TOT radical moieties within the framework could be reduced to the anionic species.

The optical band gaps of **TOT‐COF‐H** and **TOT‐COF‐L** were estimated by linear extrapolation of the absorption onsets in Tauc plots derived from Kubelka–Munk‐transformed diffuse reflectance data (Figures 2a and S14). The optical band gaps were determined to be *E*_g_ = 1.09 eV and *E*_g_ = 1.12 eV for **TOT‐COF‐H** and **TOT‐COF‐L**, respectively, positioning them among the narrowest band gaps reported to date for neutral COFs (see Table S6). These narrow band gaps indicate a reduced energy separation between the valence and conduction bands. The electronic structure of the **TOT‐COF** was subsequently investigated via density functional theory (DFT) using the PBE and PBE0 functionals (computational details are provided in the Supporting Information). The TOT‐COF structure was optimized both as a freestanding monolayer and as a bulk crystalline phase with a pdAA stacking arrangement. The calculated band structure of the monolayer exhibits flat bands near the Fermi level, and a magnetic coupling within the plane is expected to be negligible because of the large separation between radical units (Figure S20b). In the bulk structure, the antiferromagnetic and ferromagnetic configurations are nearly isoenergetic and exhibit band gaps of 1.26 and 1.20 eV, respectively, in excellent agreement with the experimental values. Each formula unit hosts a single S = 1/2 spin, with the magnetic moment primarily localized on the TOT moiety (Figures 2c, S21, and S22). Analysis of the band structure reveals substantially greater dispersion along the interlayer direction (Figure 2b), indicating that charge transport likely occurs through out‐of‐plane pathways enabled by one‐dimensional π‐stacking columns between the TOT radical units.

**FIGURE 2:**
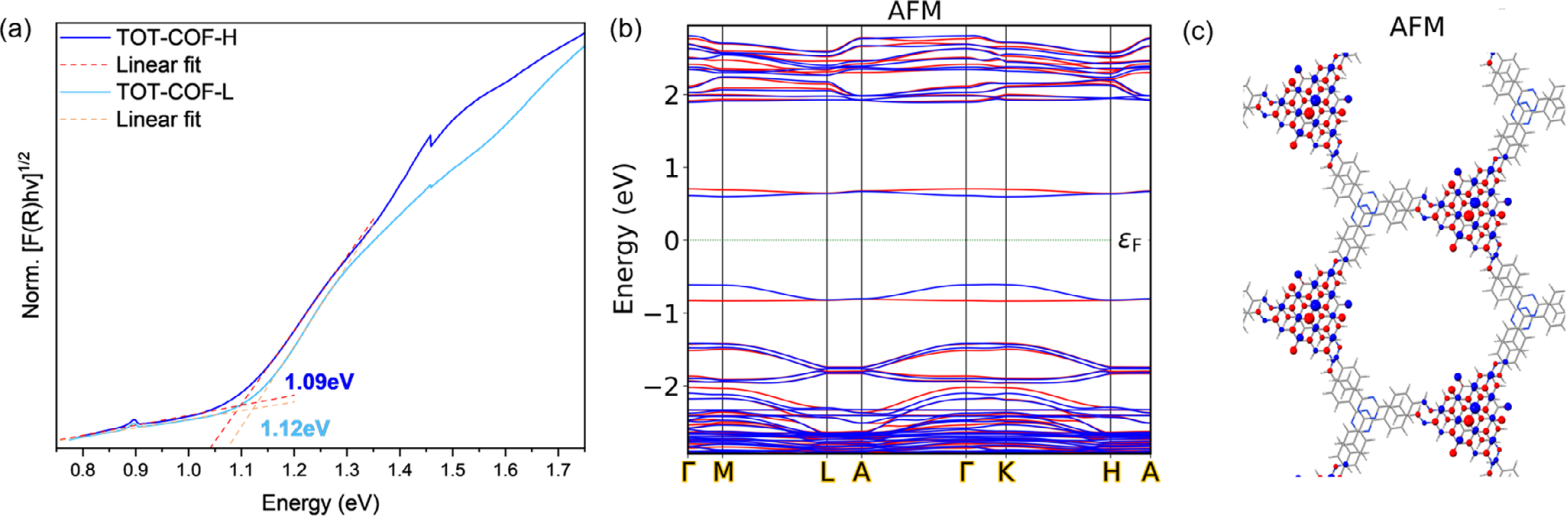
(a) Normalized Tauc plots of the Kubelka–Munk‐transformed diffuse‐reflectance data for TOT‐COF‐H and TOT‐COF‐L. Dashed lines indicate linear fits to the absorption onsets. (b) Band structure and (c) spin density of bulk TOT‐COF in the antiferromagnetic state with parallel‐displaced AA stacking, computed using the PBE0 functional. Red and blue denote opposite spin polarizations.

### 2.5 Electrical Conductivity of TOT‐COFs

Electrical conductivities of **TOT‐COF‐H** and **TOT‐COF‐L** were determined on pressed pellets using a four‐probe van der Pauw configuration (Figures 3a,b, S15, S16, and Tables S3–S5). The measured room‐temperature conductivities were found to be *σ*_RT_ = (1.17 ± 0.06) × 10^−4^ and (1.5 ± 0.8) × 10^−5^ S cm^−1^ for **TOT‐COF‐H** and **TOT‐COF‐L**, respectively (see Supporting Information). Activation energies were estimated from the temperature dependence of the pellet resistivity in the same configuration (Figure 3c), yielding values of 241 and 263 meV for **TOT‐COF‐H** and **TOT‐COF‐L**, respectively, which are similar values than those reported for other TOT‐based systems [24]. The Seebeck coefficients follow the same trend as the electrical conductivity measurements, further confirming the highest intrinsic electronic conductivity for **TOT‐COF‐H** (Figures S17a and S18). PXRD patterns of the pressed pellets after electrical measurements display the characteristic diffraction peaks of each material, indicating that the structural integrity of **TOT‐COF** is preserved after pelletization (Figure S19). The increase in the (001)/(100) peak intensity ratio is attributed to the preferential alignment of the COF layers perpendicular to the compression axis during pellet pressing [39]. Notably, the π–π stacking reflection of the pelletized materials is relatively more intense for **TOT‐COF‐H** than for **TOT‐COF‐L**, suggesting a higher degree of interlayer ordering and stronger electronic coupling between the stacked TOT radical units, which likely contributes to its higher electrical conductivity. Therefore, although both materials exhibit spin densities per unit cell of the same order of magnitude, the higher conductivity of **TOT‐COF‐H** is likely associated with its greater structural order, which may facilitate more effective orbital overlap between the TOT radical units.

**FIGURE 3:**
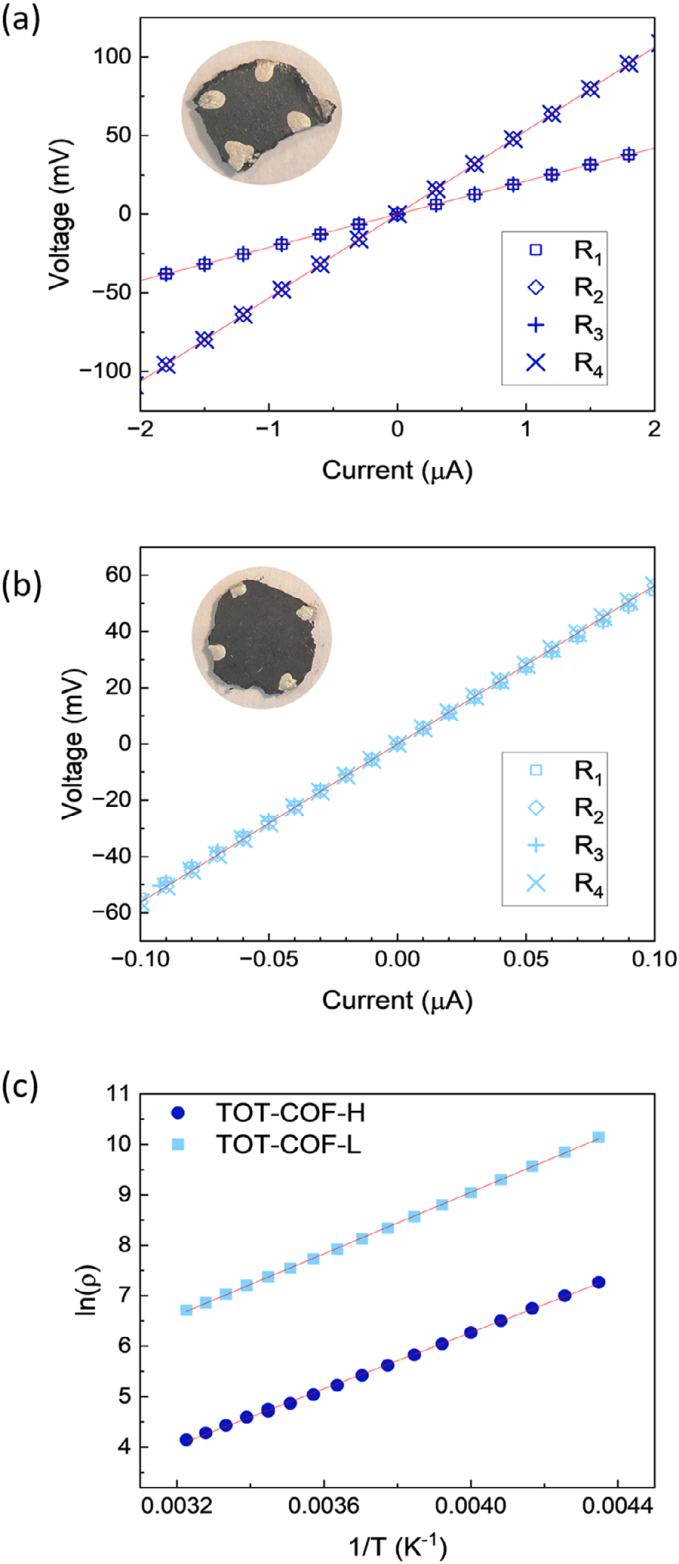
Current‐voltage (I‐V) characteristics of pressed pellets of (a) **TOT‐COF‐H**, and (b) **TOT‐COF‐L** (inset shows photographs of the pressed pellets with silver‐paste contacts). (c) Arrhenius plots derived from temperature‐dependent four‐probe conductivity measurements (red lines show linear fits).

It is worth noting that the room‐temperature conductivity of **TOT‐COF‐H** is among the highest reported for neutral, non‐doped COFs (see Table S6). In addition, in contrast to closed‐shell systems such as trioxaazatriangulene‐based COFs [10, 38, 39], which require chemical doping, the use of open‐shell TOT building blocks enables the direct incorporation of free charge carriers into the framework. Collectively, these results demonstrate that incorporating delocalized neutral π‐radicals is a promising strategy for the design of intrinsically conductive COFs without the need for extrinsic doping.

## 3 Conclusions

In summary, we have developed a synthetic strategy for semiconducting COFs by incorporating highly delocalized π‐radical TOT building blocks to enhance electrical conductivity. The synthetic approach based on the use of amino‐functionalized TOT derivatives results in a highly crystalline COF (**TOT‐COF‐H**) with an electrical conductivity one order of magnitude higher than that of the less crystalline material (**TOT‐COF‐L**). The reported conductivity (*σ*_RT_ = 1.2 × 10^−4^ S cm^−1^) is among the highest reported for neutral, non‐doped COFs. Band structure calculations reveal that charge transport primarily originates from out‐of‐plane transport pathways facilitated by uniform π‐π stacking between the TOT radical moieties. This study presents an effective approach for incorporating stable delocalized neutral radicals into COFs, enabling the design of intrinsically conductive, porous, and crystalline organic frameworks for potential applications in electronics, spintronics, and energy storage. Future work will focus on expanding the TOT‐COF family by using diverse building blocks and linkages to investigate their influence on conductivity and spin delocalization, as well as exploring their potential as electrode materials for energy storage applications.

## Author Contributions

**Paula Escamilla**: investigation, writing – original draft, writing – review and editing, methodology. **Sara Trigo‐Pérez**: investigation, methodology. **Rafael Ramos**: investigation, methodology, writing – review and editing. **Ricardo Ortiz**: investigation, methodology, software. **Manuel Vilas‐varela**: investigation, methodology. **Diego Peña**: investigation, methodology, supervision, writing – review and editing. **Manuel Melle‐franco**: investigation, methodology, supervision, writing – review and editing. **Francisco Rivadulla**: writing – review and editing, supervision, methodology, investigation. **Manuel Souto**: conceptualization, funding acquisition, investigation, writing – original draft, writing – review and editing, methodology, supervision.

## Conflicts of Interest

The authors declare no conflicts of interest.

## Supporting information




**Supporting File**: anie74125‐sup‐0001‐SuppMat.pdf.

## Data Availability

The data that support the findings of this study are available in the Supporting Information of this article.
