# Supplementary material for: Semiconducting Covalent Organic Frameworks Based on Spin‐Delocalized Trioxotriangulene Neutral Radicals
Source: Angew Chem Int Ed Engl. 2026 Aug 10;65(41):e6104022. doi: 10.1002/anie.6104022 (PMC13638113; doi:10.1002/anie.6104022)
Supplement: Supplementary file 1 [file ANIE-65-e6104022-s001.pdf]

Supporting Information

Semiconducting Covalent Organic Frameworks Based on Spin-Delocalized Trioxotriangulene Neutral Radicals

Paula Escamilla,[a],* Sara Trigo,[a] Rafael Ramos,[a] Ricardo Ortiz,[b] Manuel Vilas-Varela,[a] Diego Peña,[a,c] Manuel Melle-Franco,[b] Francisco Rivadulla,[a] Manuel Souto[a,c],*

[a]*Centro Singular de Investigación en Química Biolóxica e Materiais Moleculares (CiQUS), Departamento de Química Física, Universidade de Santiago de Compostela, 15782 Santiago de Compostela, Spain*

[b]*Department of Chemistry, CICECO-Aveiro Institute of Materials, University of Aveiro, 3810-393 Aveiro, Portugal*

[c]*Oportunius, Galician Innovation Agency (GAIN), 15702 Santiago de Compostela, Spain*

Table of contents

1. **General Information** S3
2. **Synthetic procedures** S5
3. **Physicochemical characterization of TOT-COF** S9
4. **Electron spin resonance (ESR)** S15
5. **Cyclic voltammetry** S17
6. **Electrical conductivity measurements** S18
7. **Computational details** S23
8. **References** S25

1. General Information

All reagents and solvents employed in the synthesis were of high-purity grade and were purchased from Sigma-Aldrich Co. or TCI.

^{1}H, and ^{13}C NMR experiments were carried out using a Varian Mercury 300MHz NMR spectrometer. Chemical shift values are reported in ppm with the solvent resonance as the internal standard ($CHCl_3$: δ = 7.26 ppm for ^{1}H; DMSO: δ = 2.5 ppm for ^{1}H).

Solid-state NMR spectra were measured at 298K in a Bruker NEO-750 spectrometer (750 MHz proton frequency) equipped with a Varian T3 solid triple resonance probe 1H/X/Y that uses zirconia rotor of an outer diameter of 3.2 mm. The probe was adapted to the Bruker spectrometer by a A2B conversion kit (Revolution NMR, LLC). Solid-state ^{13}C NMR spectra were recorded on a Bruker NEO 750 spectrometer using a 3.2 mm double-resonance magic angle spinning (MAS probe). 1D ^{13}C-CP-MAS spectrum (sequence "hC.cp" of the Bruker library) was measured with an inter-scan delay (d1) of 1.5 s, 12000 scans and a MAS rate was 20 kHz. Cross-polarization (CP) was applied during 1 ms with a constant carbon field strength of 63.1 kHz. During CP the power on the ^{1}H nucleus was linearly ramped from 70% to 100% with a peak field strength of 59.0 kHz. Heteronuclear proton decoupling during the FID acquisition was performed with Spinal-64 with a proton field strength of 79.4 kHz. Carbon-13 chemical shifts were referenced externally to the methylene signal of crystalline α-form of glycine at 43.5 ppm.

MALDI-TOF-MS were performed on an Autoflex Bruker spectrometer using negative mode.

Scanning electron microscopy (SEM) images were recorded using a FESEM equipment (ultra plus, Zeiss, Germany).

High-resolution transmission electron microscopy analysis was performed on JEOL JEM F200CF-HR Microscope (200KeV) with a cold field emission electron gun and HR objective lens.

Powder X-ray diffraction (PXRD) patterns were recorded using a Rigaku MiniFlex 600-C X-ray diffractometer using Cu Kα radiation (λ = 1.54056 Å). The X-ray tube was operated at a voltage of 40 kV and a current of 15 mA. Data were collected in the ca. $2^{o} \leq 2\theta \leq 40^{o}$ range.

Gas sorption measurements were recorded ex-situ on a Micromeritics 3Flex apparatus. The samples were degassed overnight at 100 ºC and 10^{-6} Torr prior to analysis. BET surface values were calculated from the N_2 isotherms using BETSI[1] and pore size distributions were obtained using the non-local density functional theory (NLDFT) method.

ESR measurements. ESR spectra were recorded using a Bruker EMX spectrometer equipped with a TE101 rectangular cavity and a Bruker Magnettech 5000 instrument. Experimental parameters, including sample amount, positioning within the cavity, microwave power, number of scans, conversion time, and modulation amplitude, were optimized to maximize the signal-to-noise ratio while maintaining signal linearity and avoiding saturation. Typical operating conditions were as follows: microwave power, 1 mW; modulation amplitude, 0.5 G; receiver gain, 1×10^{5}; time constant, 5.12 ms; and conversion time, 20.48 ms. Magnetic susceptibility measurements were performed using a MPMS3 SQUID-VSM Magnetometer (7 Teslas) and a magnetic field of H = 4000 Oe.

Thermogravimetric analysis (TGA) of the powder samples was conducted employing a TA Instruments Q5000 IR thermobalance. The TGA measurements involved a general heating profile ranging from 25 to 900 °C, with a heating rate of 5 °C min^{-1} under an N_2 atmosphere using a gas flow rate of 25 mL min^{-1}.

FT-IR measurements were conducted employing a PerkinElmer Spectrum Two spectrometer equipped with Attenuated Total Reflection (ATR) capability. The dried powders were positioned on the ATR window for analysis within the wavenumber range of 400 to 4000 cm^{-1}.

The UV-vis-NIR absorption and diffuse reflectance spectra of the samples were measured using a Lambda 950 dual-beam spectrometer (PerkinElmer) and Reflectance FLEX Pack (Sarspec). The diffuse reflectance spectra are reported as the Kubelka-Munk transform, where $F(R) = (1-R)^2 /2R$. The indirect optical band gaps of these materials were determined from respective Tauc plots.

Cyclic voltammetry (CV) and Differential Pulse Voltammetry (DPV) were performed on an Autolab Metrohm VIONIC potentiostat/galvanostat (powered by INTELLO). All electrochemical experiments were conducted in a conventional three-electrode configuration, consisting of a glassy carbon working electrode, a platinum wire counter electrode, and a silver reference electrode. Measurements were carried out at room temperature under an inert atmosphere. A 0.1 M solution of tetrabutylammonium perchlorate (Bu_4NClO_4) in THF was used as the electrolyte. CV measurements were recorded at scan rates of 0.1, 0.3 and 0.5 $V{\cdot}s^{-1}$ and ferrocene (Fc) was used as an internal standard. Experimental parameters were adjusted as appropriate for each measurement.

2. Synthetic procedures

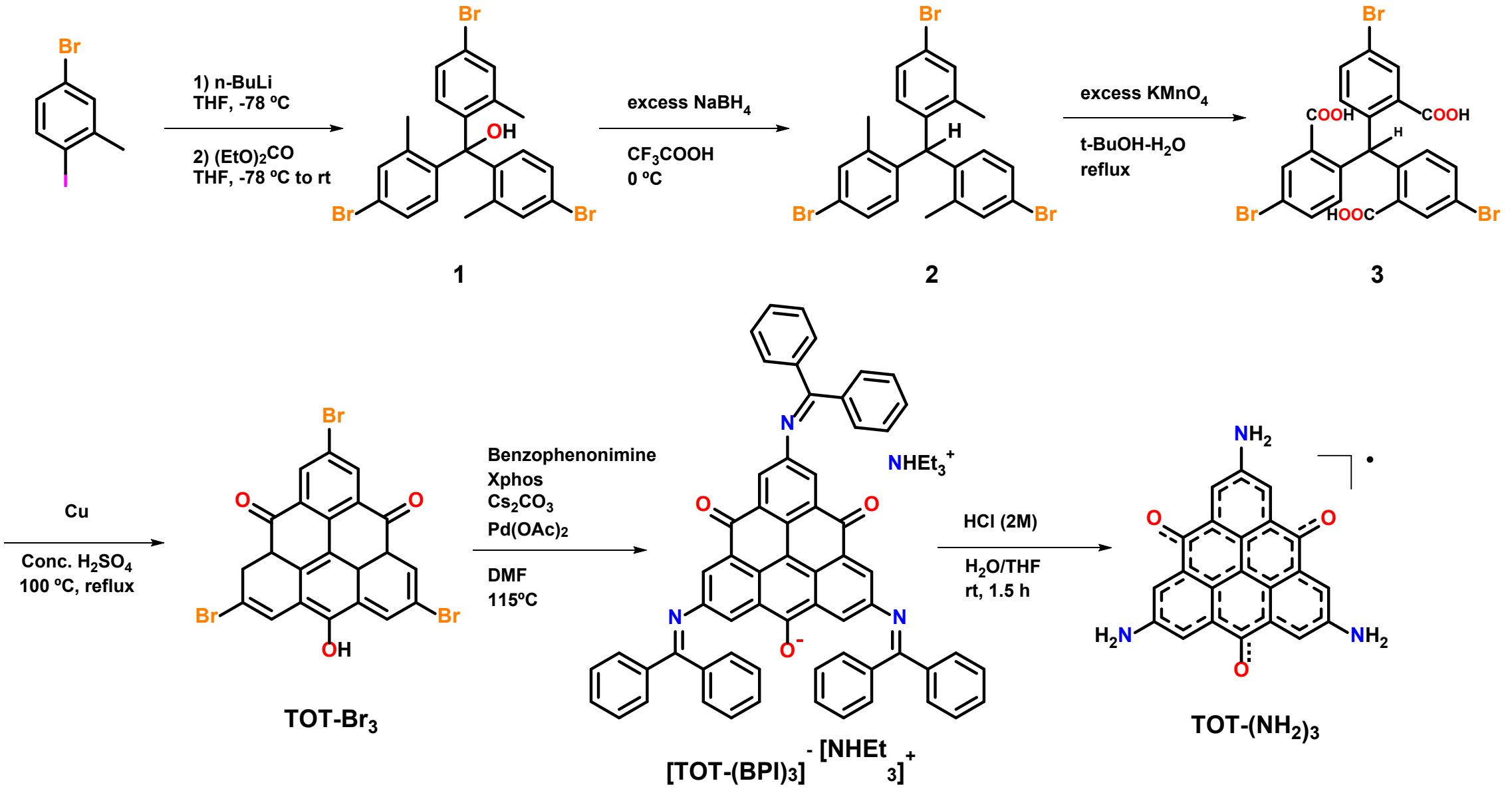

Scheme 1. Synthetic route of **TOT-(NH$_2$)$_3$**.

Compounds **1** to **3** were synthesized according to the literature with minor modifications.[2]

Tris(2-methyl-4-bromophenyl)methanol (**1**)**.** To a mixture of 2-iodo-5-bromotoluene (5 mL, 35 mmol) and dry THF (150 mL) in a 250 mL round-bottomed flask, n-BuLi (2.5 M hexane solution, 14.4 mL, 36 mmol) was added dropwise at –78 °C. Diethyl carbonate (1.4 mL, 11.5 mmol) was then added to the mixture, and the reaction mixture was allowed to warm to room temperature and stirred for 1 h. The mixture was poured into water (50 mL) and extracted with ethyl acetate (100 mL). The organic phase was washed with saturated NaCl aqueous solution (25 mL), dried over anhydrous Na_2SO_4, and concentrated under reduced pressure. The resulting residue was purified by silica gel column chromatography using hexane/ethyl acetate (95/5) as the eluent to afford a white powder (2.6 g, 42%). ^{1}H NMR (300 MHz, $CDCl_3$): δ (ppm): 2.16 (s,9H), 2.80 (s, 1H), 6.55 (d, J = 8.5 Hz, 3H), 7.19 (d, J = 8.6, 2.2 Hz, 3H) and 7.38 (d, J = 2.1 Hz, 3H).

Tris(2-methyl-4-bromophenyl)methane (**2**)**.** In a 150 mL round-bottomed flask, **1** (1.75 g, 3.2 mmol) was dissolved in CF_3COOH (75 mL). Then, $NaBH_4$ (2.2 g, 58.2 mmol) was added portion wise to the solution in an ice bath, and the reaction mixture was stirred at this temperature for 3 h. The mixture was then neutralized with a saturated NaOH aqueous solution and extracted with ethyl acetate (100 mL). The organic phase was washed with saturated NaCl aqueous solution (25 mL), dried over anhydrous Na_2SO_4, filtered and concentrated under reduced pressure. The resulting residue was purified by silica column chromatography using hexane as the eluent to afford a white powder (1.4 g, 82%). ^{1}H NMR (300 MHz, $CDCl_3$): δ (ppm) = 2.08 (s, 9H), 5.46 (s, 1H), 6.50 (d, J = 8.5 Hz, 3H), 7.20 (d, J = 8.3, 2.1 Hz, 3H), 7.33 (d, J = 2.3 Hz, 3H).

Tris(2-carboxyl-4-bromophenyl)methane (**3**)**.** In a 1-L round-bottomed flask, **2** (1.4 g, 2.68 mmol) and $KMnO_4$ (13.72 g, 86 mmol) were suspended in a mixture of *t*-BuOH (100 mL) and water (100 mL). The reaction mixture was refluxed for 48 h. After cooling to room temperature, the insoluble material was removed by filtration and washed with ethyl acetate. The filtrate was acidified with 2 M HCl aqueous solution (85 mL) and

extracted with ethyl acetate (150 mL). The organic phase was washed with saturated NaCl aqueous solution (25 mL), then dried over Na_2SO_4, filtered, and concentrated under reduced pressure. The resulting residue was washed with CH_2Cl_2 (5 mL) to afford **3** (1.20 g, 73%) as a white powder. ^{1}H NMR (300 MHz, DMSO-d$_6$): δ (ppm) = 6.74 (d, *J* = 8.3 Hz, 3H), 7.64 (d, *J* = 8.4, 2.3 Hz, 3H), 7.79 (s, 1H), 7.93 (d, *J* = 2.2 Hz, 3H) ppm.

2,6,10-Tribromo-4,8-dioxo-4H,8H-dibenzo[*cd,mn*]pyren-12-ol (TOT-Br$_3$). In a 25-mL round-bottomed flask, **3** (1.2 g, 1.96 mmol) and copper (0.165 g, 2.6 mmol) were dissolved in concentrated H_2SO_4 (17 mL). The reaction mixture was stirred at 100 °C for 48 h. The mixture was then hot-filtered and poured into ice–water. The resulting precipitate was collected by centrifugation and washed with water, THF, and acetone to afford **TOTBr$_3$** (525 mg, 48%) as a blue powder. ^{1}H NMR (300 MHz, DMSO-d$_6$): δ (ppm) = 8.66 (s, 6H) ppm; MALDI-TOF (negative mode) *m/z*: 559.

2,6,10-Tris((diphenylmethylene)amino)-12-hydroxydibenzo[cd,mn]pyrene-4,8-dione ([TOT-(BPI)$_3$]$^-$[NHEt$_3$]$^+$). In a dried 50-mL round-bottom flask, **TOT-Br$_3$** (200 mg, 0.35 mmol), $Pd(OAc)_2$ (42 mg, 0.18 mmol), 2-dicyclohexylphosphino-2′,4′,6′-triisopropylbiphenyl (XPhos) (127 mg, 0.27 mmol), and Cs_2CO_3 (842 mg, 2.58 mmol) were mixed under argon atmosphere. Then, dry DMF (35 mL) and benzophenone imine (0.58 mL, 3.45 mmol) were added, and the reaction mixture was heated under reflux at 115 °C for 3 days. After cooling to room temperature, the reaction mixture was filtered and concentrated under reduced pressure to yield a black solid, which was washed with diethyl ether and dried. The crude product was purified by silica gel column chromatography using a gradient of 2% MeOH in CH_2Cl_2:NEt_3 (99:1) as the eluent. The resulting residue was dissolved in CH_2Cl_2 and extracted with a 0.5 M aqueous thiourea solution containing 1% NEt_3 (25 mL). The organic phase was washed with aqueous NaCl solution with 1% NEt_3 (25 mL) and dried over anhydrous Na_2SO_4, filtered and concentrated under reduced pressure to afford **[TOT-(BPI)$_3$]$^-$[NHEt$_3$]$^+$** as a dark green solid (180 mg, 59%). ^{1}H NMR (300 MHz, DMSO-d$_6$): δ (ppm) = 7.99 (s, 6H), 7.80 – 7.68 (m, 6H), 7.59 – 7.45 (m, 9H), 7.34 – 7.24 (m, 8H), 7.24 – 7.14 (m, 6H), 2.96 (d, *J* = 7.5 Hz, 9H), 1.12 (t, *J* = 7.2 Hz, 12H); ^{13}C NMR (75 MHz, DMSO-d$_6$): δ (ppm) = 180.37, 168.42, 144.65, 139.68, 136.33, 131.34, 129.61, 129.31, 129.10, 128.87, 128.77, 127.34, 125.20, 46.25, 9.66. MALDI-TOF (negative mode) *m/z*: 857 (M^- - $NHEt_3$).

2,6,10-triamino-12-hydroxydibenzo[*cd,mn*]pyrene-4,8-dione (TOT-(NH$_2$)$_3$). In a 50mL round-bottom flask, [TOT(BPI)$_3$]$^-$[NHEt$_3$]$^+$ (75 mg, 0.08 mmol) was dissolved in a H_2O/THF mixture (26 mL, 1:1 v/v). To the resulting green solution was added 2 M HCl (0.10 mL, 0.20 mmol). The reaction mixture was stirred at room temperature and under ambient conditions for 1.5 h. The resulting suspension was washed with H_2O and acetone by centrifugation. **TOT-(NH$_2$)$_3$** was isolated as a dark green powder (30 mg, ~100%). MALDI-TOF (negative mode) *m/z*: 367.

Synthesis of TOT-COF

TOT-COF-H. TOT-$(NH_2)_3$ (0.081 mmol, 30 mg), **TFPT** (0.086 mmol, 34 mg) and aqueous acetic acid (6 M, 0.39 mL) were added to a mixture of *o*-dichlorobenzene (0.39 mL) and *n*-butanol (3.5 mL) in a Pyrex tube. Then, the mixture was sonicated for 5 minutes. The tube was sealed under air and heated in an oven at 150 ºC for 3 days with a 2h ramp-up and down. After cooling to room temperature, the precipitate was collected through filtration and washed successively with DMF, THF, acetone, DCM and hexane. The resulting dark blue powder (**TOT-COF-H**) was dried at room temperature prior to further characterization (32 mg, 52%).

TOT-COF-L. $[TOT\text{-}(BPI)_3]^-[NHEt_3]^+$ (0.035 mmol, 30 mg), **TFPT** (0.035 mmol, 13.77 mg) and aqueous acetic acid (6 M, 0.43 mL) were added to a mixture of *o*-dichlorobenzene (0.43 mL) and *n*-butanol (3.9 mL) in a Pyrex tube. Then, the mixture was sonicated for 5 minutes. The tube was sealed under air and heated in an oven at 150 ºC for 3 days with a 2h ramp-up and down. After cooling to room temperature, the precipitate was collected through filtration and washed successively with DMF, THF, acetone, DCM and hexane. The resulting dark blue powder (**TOT-COF-L**) was dried at room temperature prior to further characterization (20.4 mg, 83%).

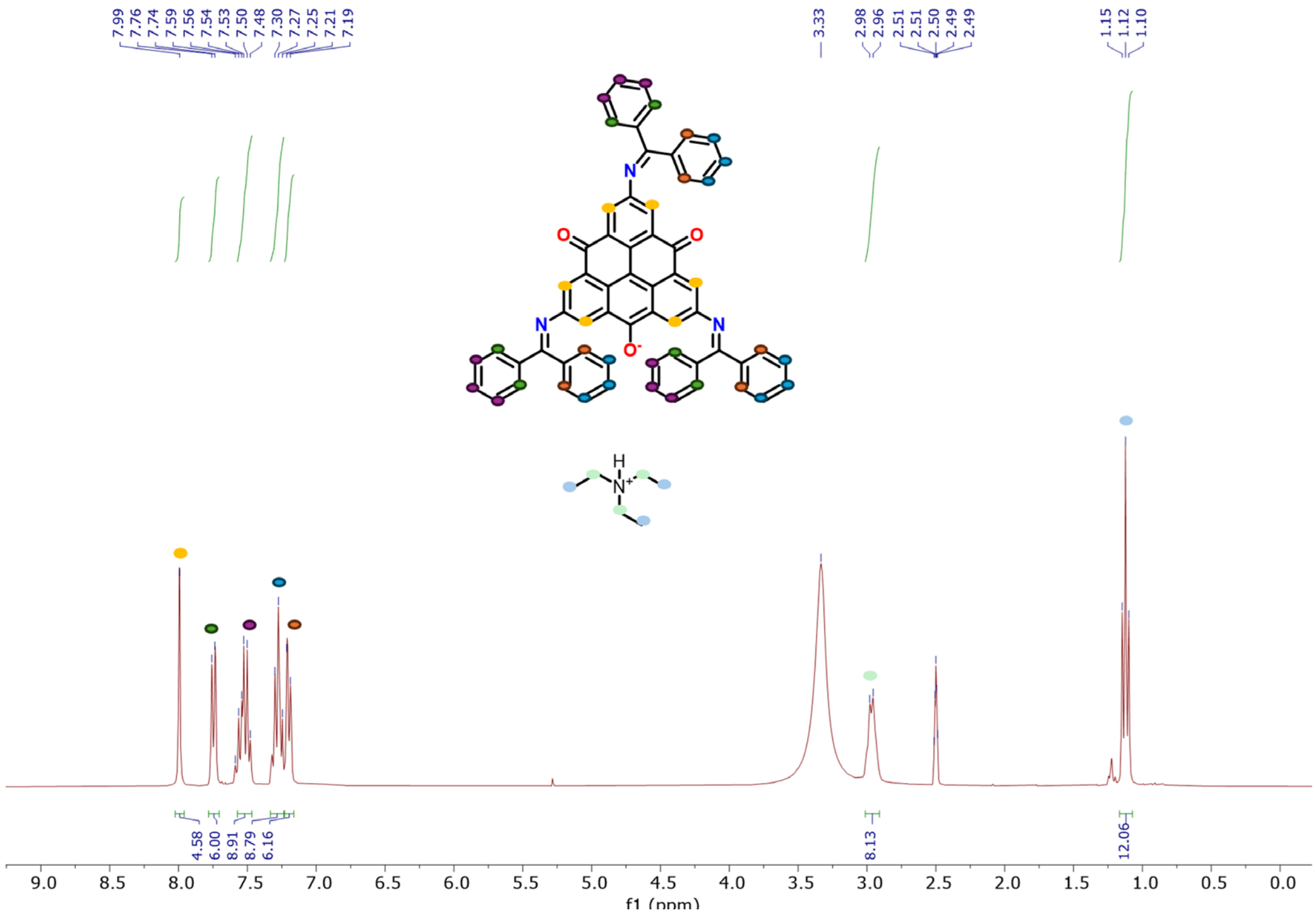

Figure S1. ^{1}H NMR spectrum of **$[TOT(BPI)_3]^-[NHEt_3]^+$** in DMSO-d6.

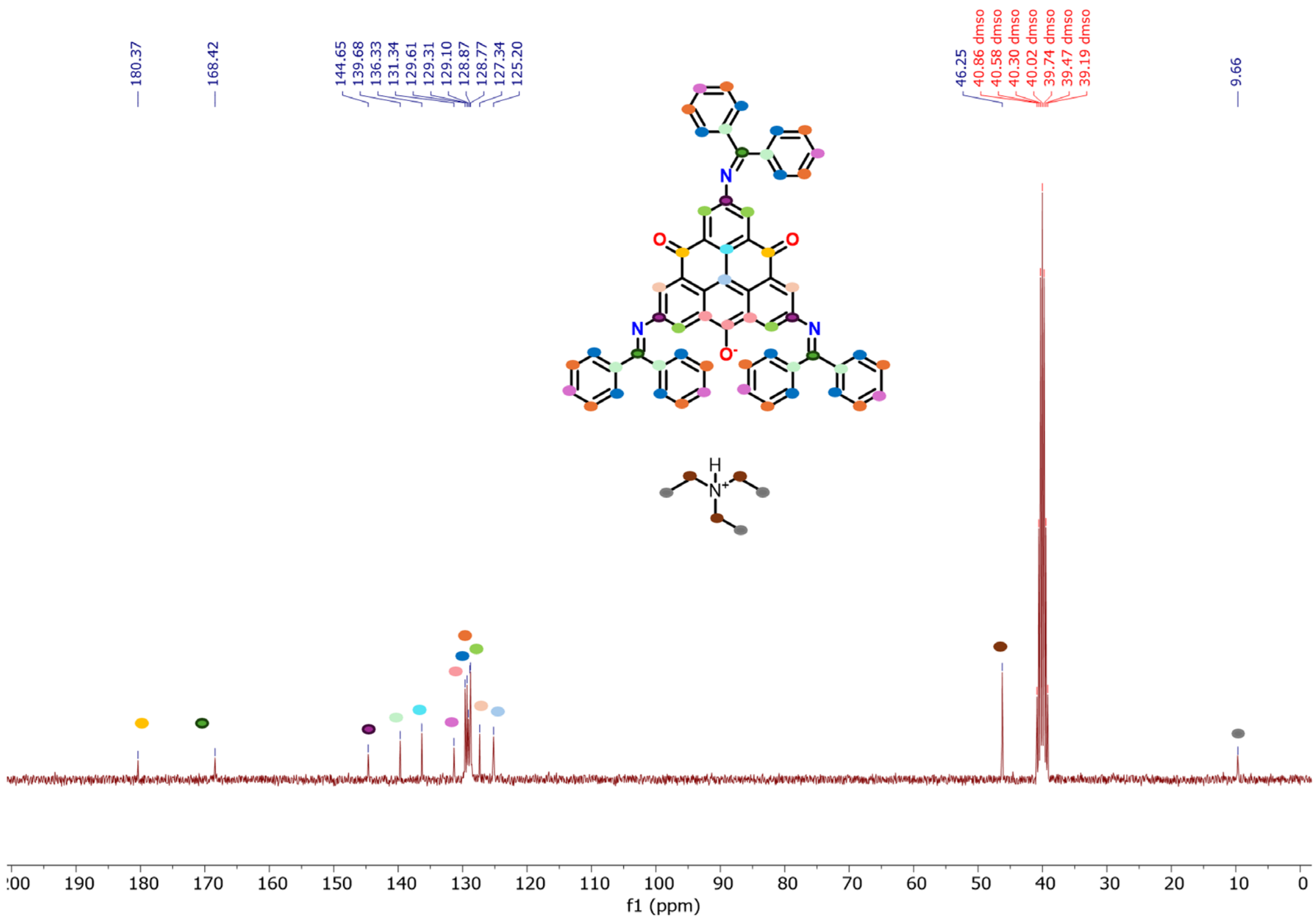

Figure S2. ^{13}C NMR spectrum of **$[TOT(BPI)_3]^-[NHEt_3]^+$** in DMSO-d6.

3. Physicochemical characterization of TOT-COF

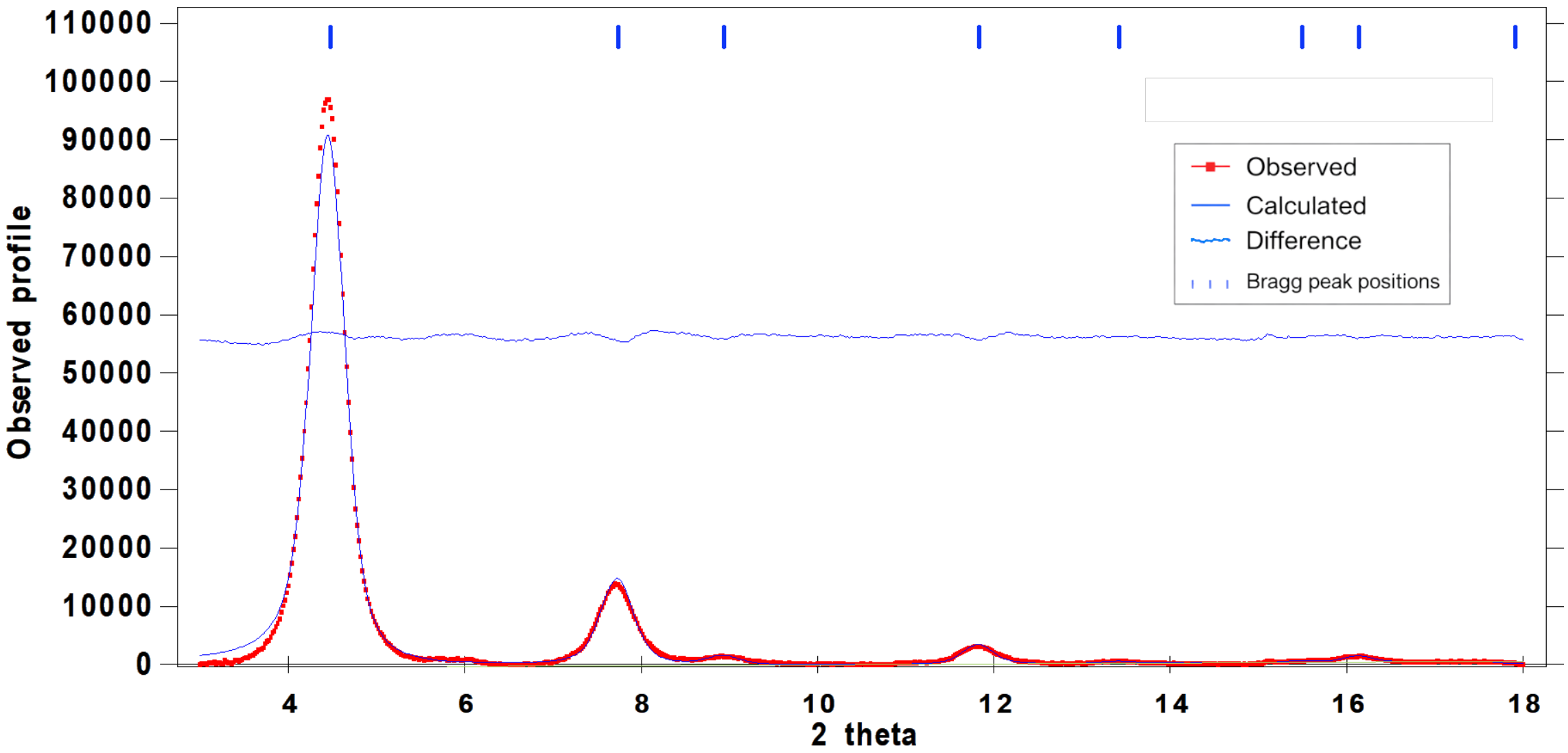

Figure S3. PXRD pattern and Pawley refinement (using DASH software) of **TOT-COF**.

Table S1. Pawley fitting and unit cell parameters.

Parameters	Refined Values
a, b [Å]	22.886
α, β (°)	90
γ (°)	120
Zero Point	0.0262
Space Group	P6
R_{wp}	12.99
R_{exp}	3.90
χ^2	11.08

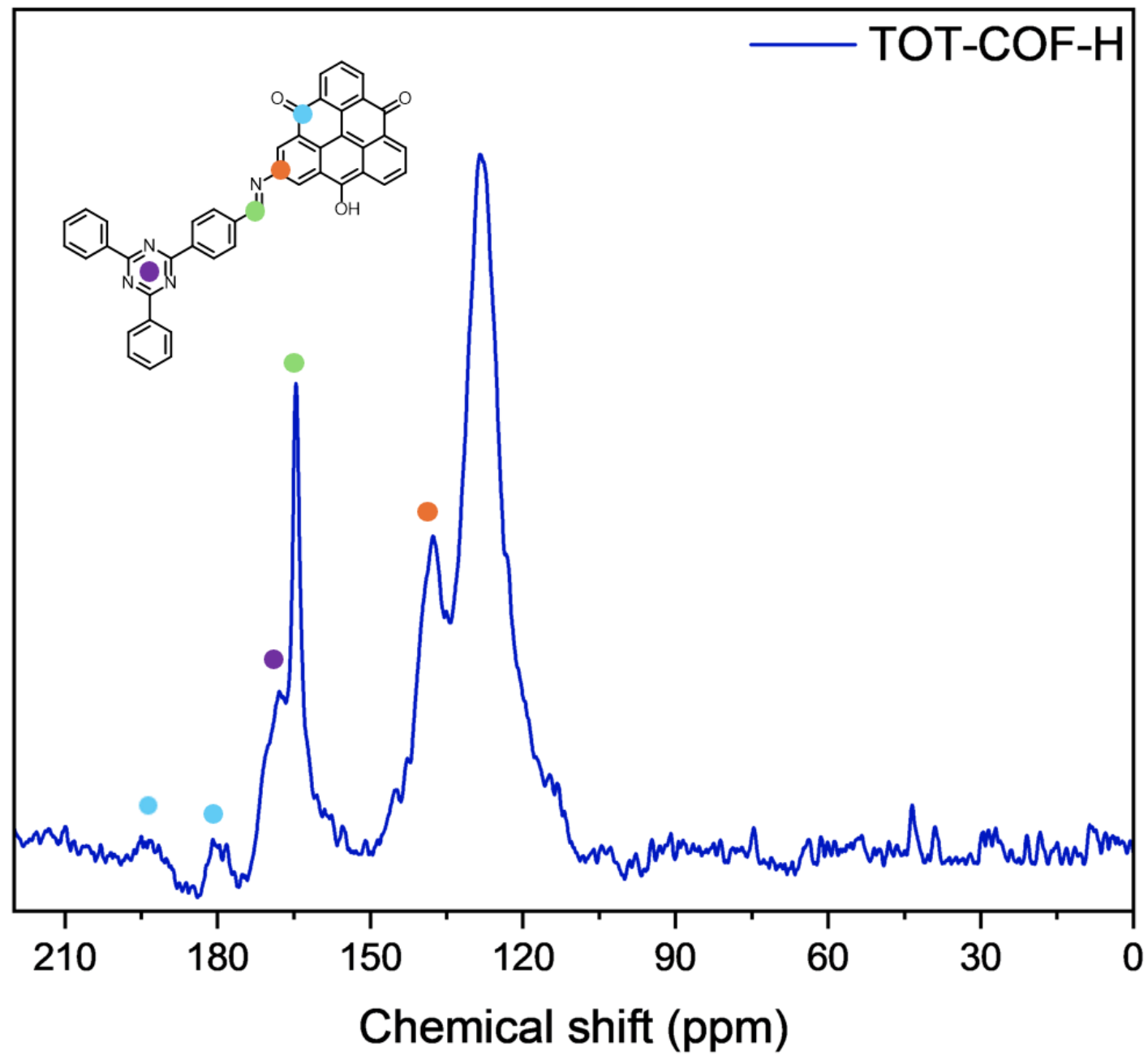

Figure S4. Solid-state cross-polarization magic-angle-spinning (CP-MAS) ^{13}C NMR spectrum of **TOT-COF-H** at room temperature.

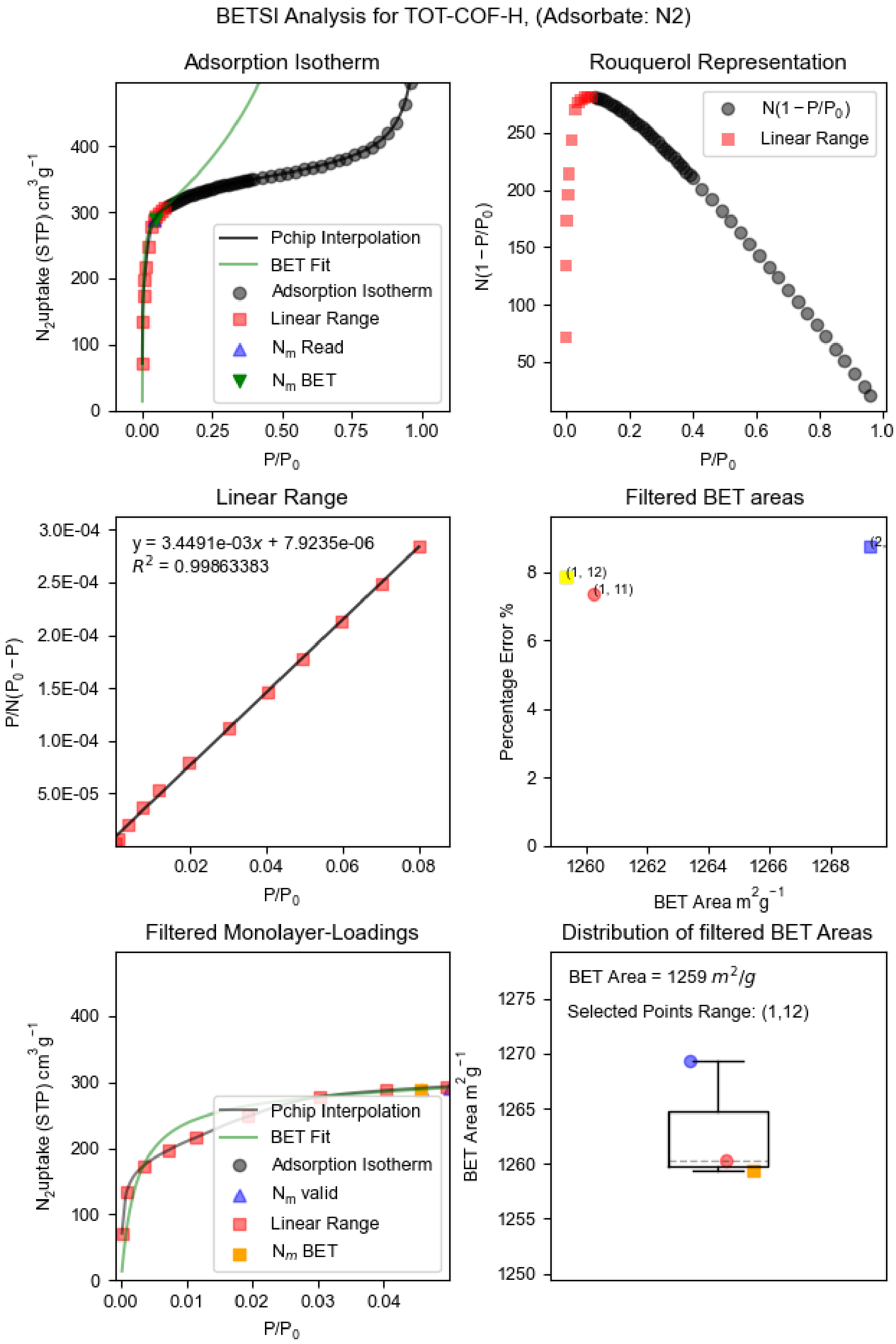

Figure S5. BETSI regression diagnostics for **TOT-COF-H**.

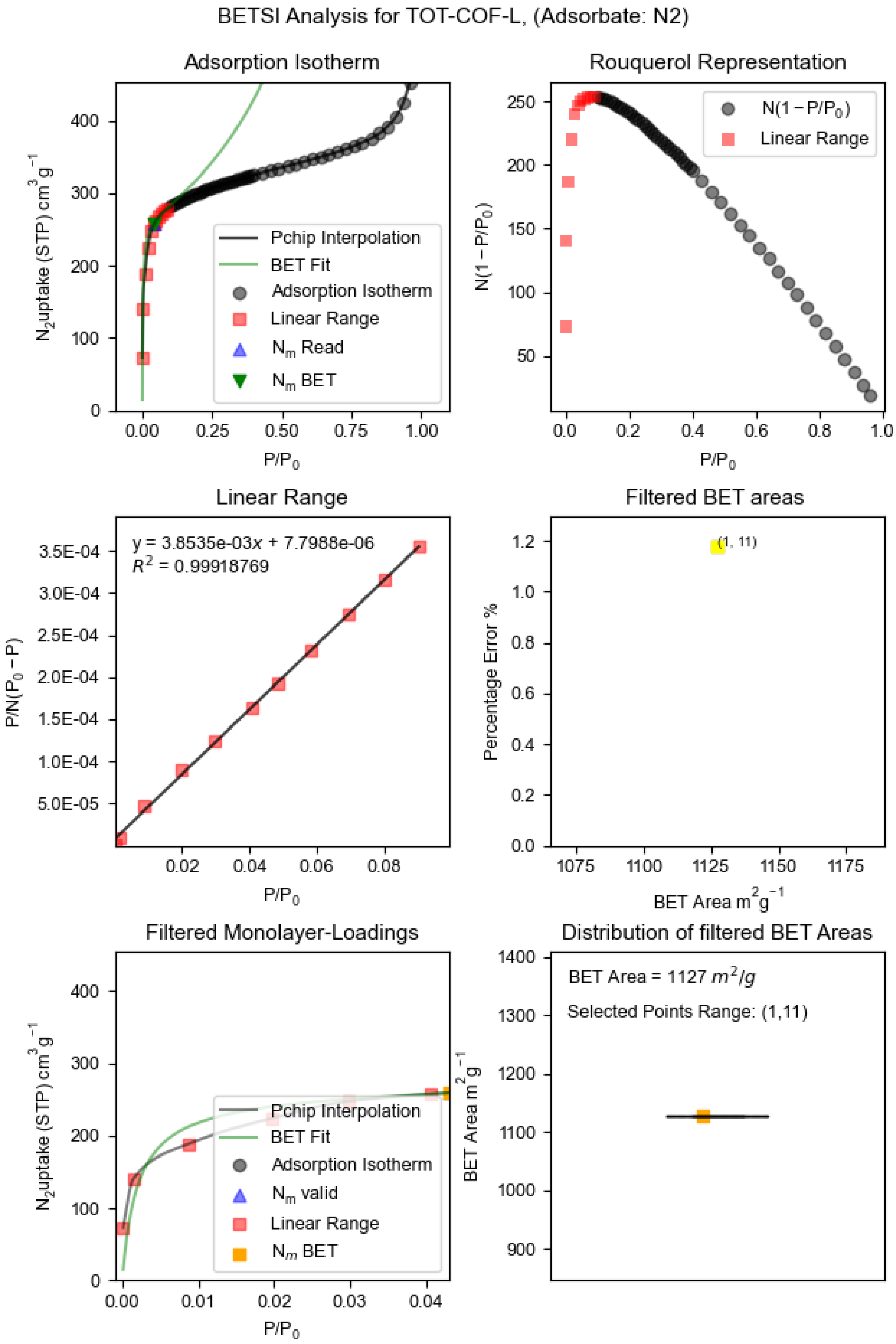

Figure S6. BETSI regression diagnostics for **TOT-COF-L**.

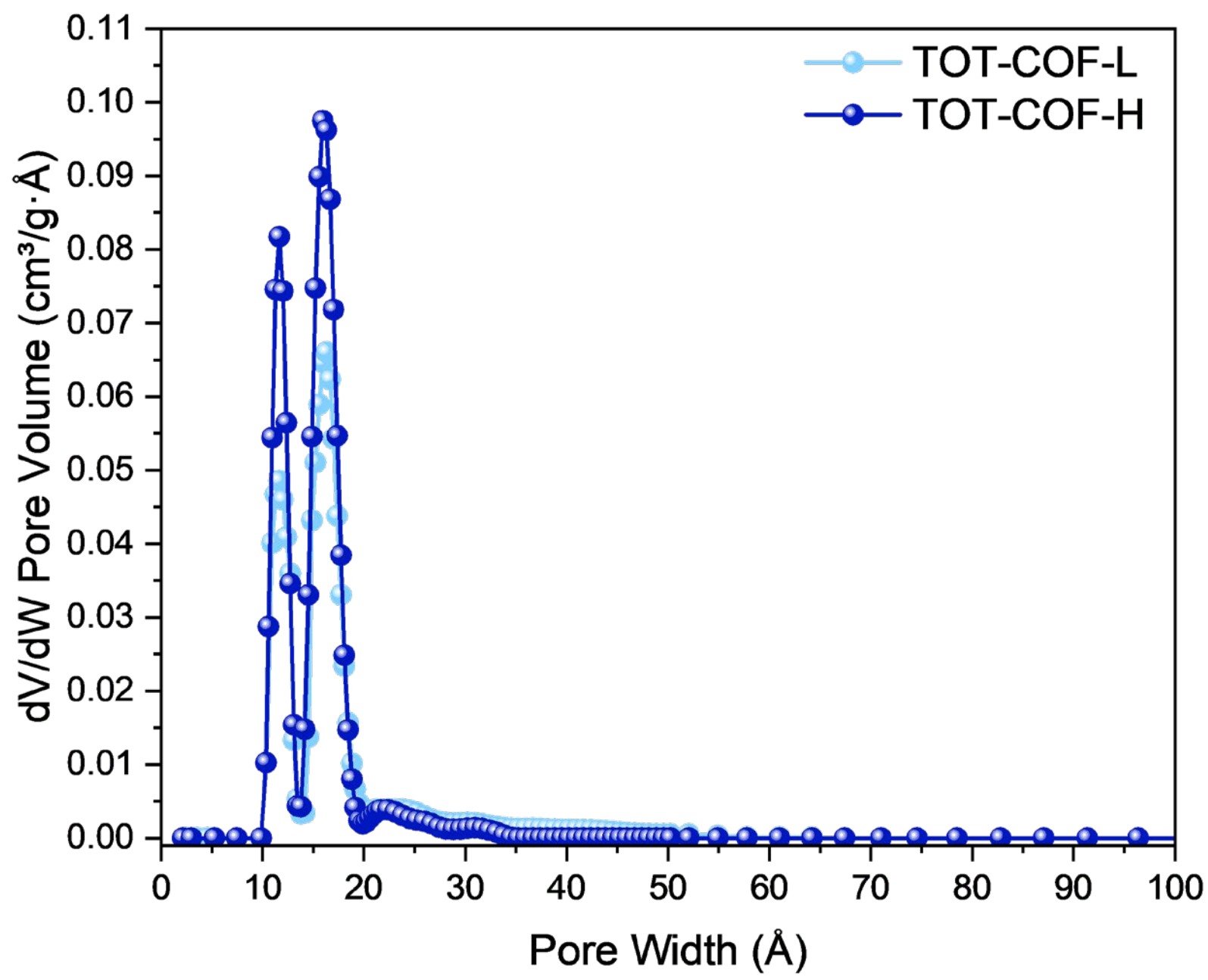

Figure S7. Pore size distribution of **TOT-COF-L** and **TOT-COF-H** using the NLDFT method.

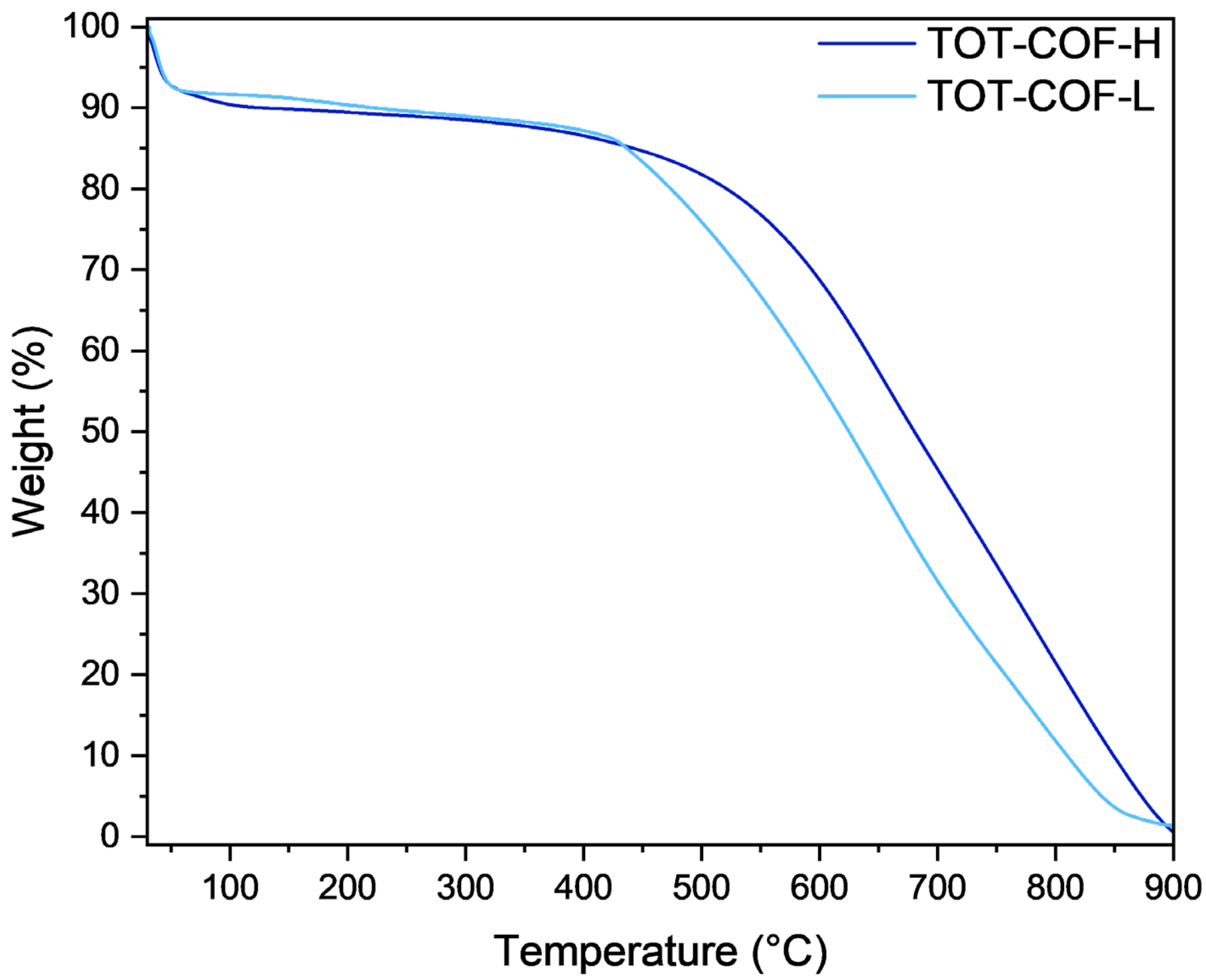

Figure S8. TGA profile of **TOT-COF-H** and **TOT-COF-L** at a heating rate of 5 °C min^{-1} under a constant stream of N_2.

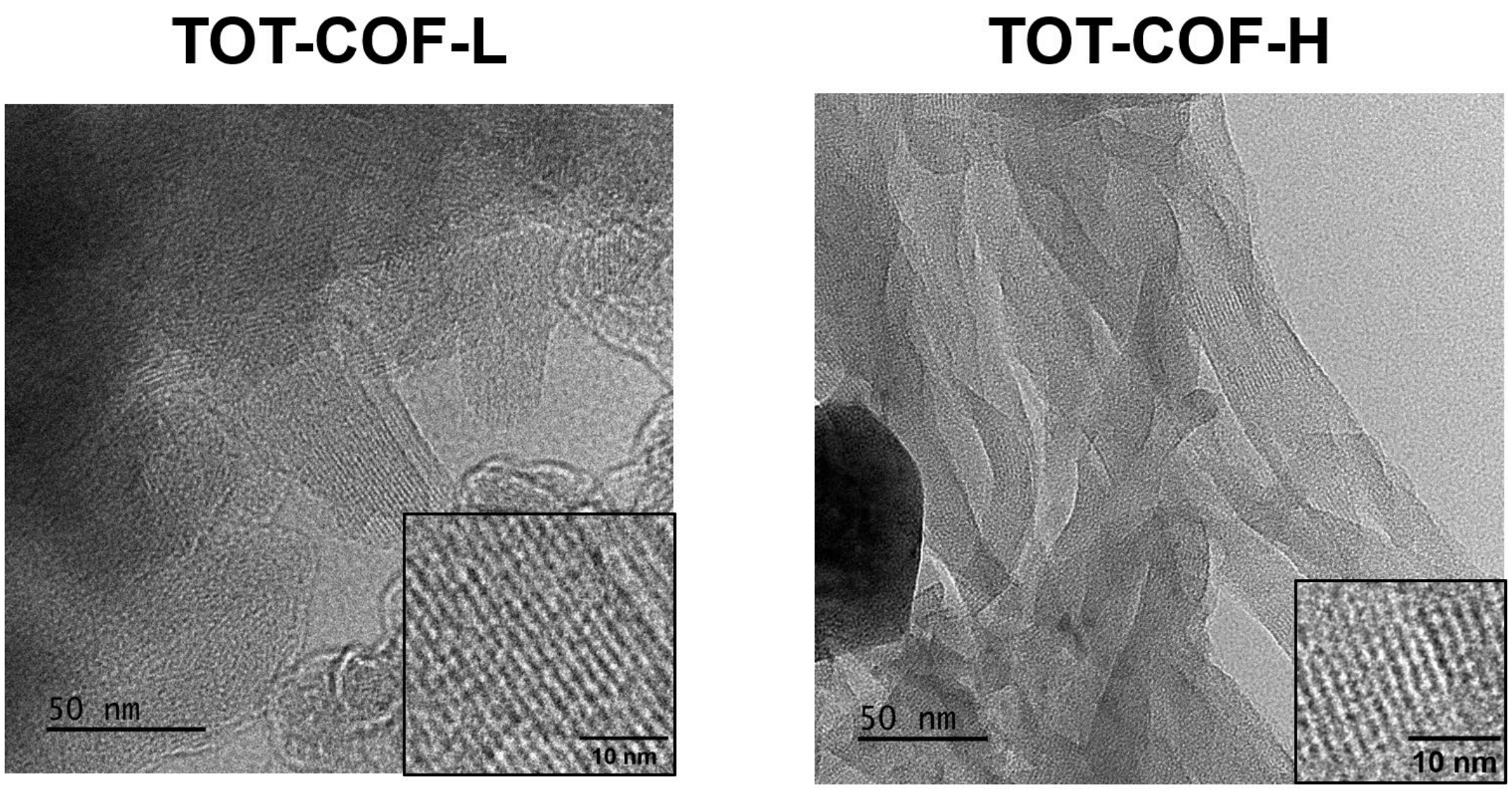

Figure S9. HRTEM images of **TOT-COF-H** and **TOT-COF-L**.

4. Electron spin resonance (ESR)

The amount of sample introduced into the ESR tube, its position in the cavity, the microwave power, number of scans, conversion time and modulation amplitude, were optimized to achieve the best signal to noise ratio while maintaining signal linearity and avoiding saturation.

Typical conditions used for the measurements reported were: microwave power of 1 mW; modulation amplitude of 0.5 G; receiver gain of 1×10^5; time constant of 5.12 ms; conversion time of 20.48 ms.

The doble integral (DI) of the ESR signal is related to the number of spins in the sample (n_s) through the following equation:

$$DI = C \times \sqrt{MWP} \times Q \times S(S+1) \times n_s$$

Where C is a constant which includes details of the experiment (time constant, spatial distribution of the microwave and modulation fields, modulation amplitude, etc), and MWP and Q are the microwave power and quality factor of the resonant cavity, respectively.[3]

Therefore, comparing the DI of a standard with that of our sample, performed under the same conditions, results in:

$$n_{s(sample)} = n_{s(std)} \frac{DI(sample)}{DI(std)}$$

To ensure the reliability of the quantification, the EPR spin counting was calibrated using two independent standard systems: (i) DPPH dispersed in quartz powder over a concentration range of 20–150 ppm, and (ii) TEMPO (2,2,6,6-tetramethylpiperidin-1-yl)oxyl dissolved in hexane at different concentrations. Both standards yielded consistent calibration curves. To minimize experimental error, all measurements were performed under identical conditions, including constant cavity filling factors, microwave power (to avoid saturation), and modulation amplitudes.

For the experiments reported in this paper, ≈1 mg of each sample was loaded into EPR tubes of 2 mm internal diameter. Thus, the loading factor of the cavity is similar in all of them. Similar conditions were used for the standard.

The quantified data in Table S1 were obtained using the corresponding molecular weight. In the case of COFs, the molecular weight of the unit cell was used.

$$N = n_{s(std)} \frac{n_{s(sample)}}{(N_A m)/M_w}$$

Table S2. Experimental and calculated values from ESR spectra to quantify the number of spin centres per molecule or unit cell.

	**m (mg)**	**M_w (g/mol)**	**Area (DI)**	**n_s**	**N**
**$[TOT(BPI)_3]^-$ $[NHEt_3]^+$**	1.9	961.16	845	6.014E+15	0.00505
**$TOT(NH_2)_3$**	1.2	367.10	133665	9.514E+17	0.484
**TOT-COF-H**	0.9	1413.41	21490	1.700E+20	0.399
**TOT-COF-L**	1.2	1413.41	48918	2.901E+20	0.681

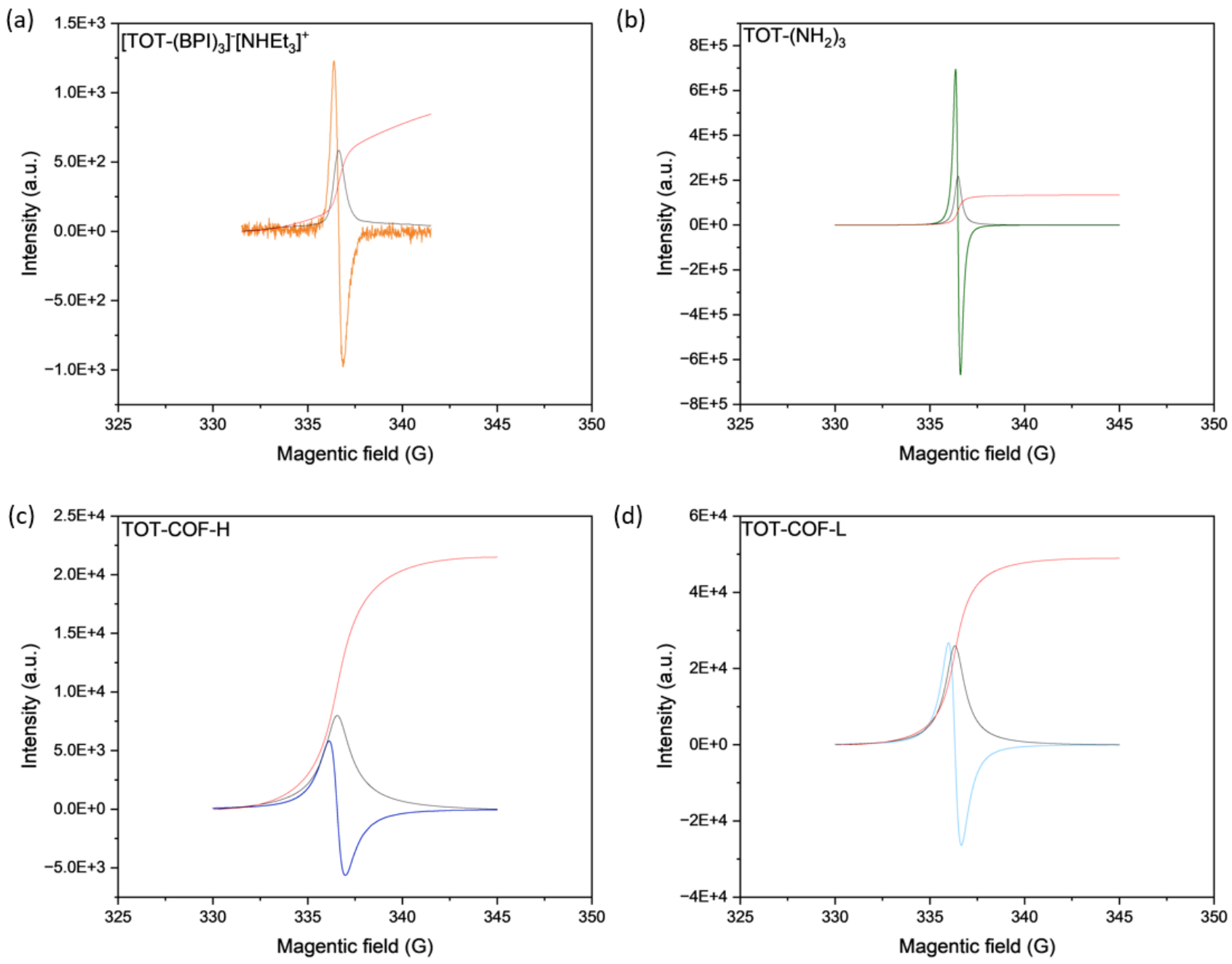

Figure S10. ESR spectra of precursors, **TOT-COF-H** and **TOT-COF-L** showing the absorption (integral of the signal, in black), and the double integral (red line).

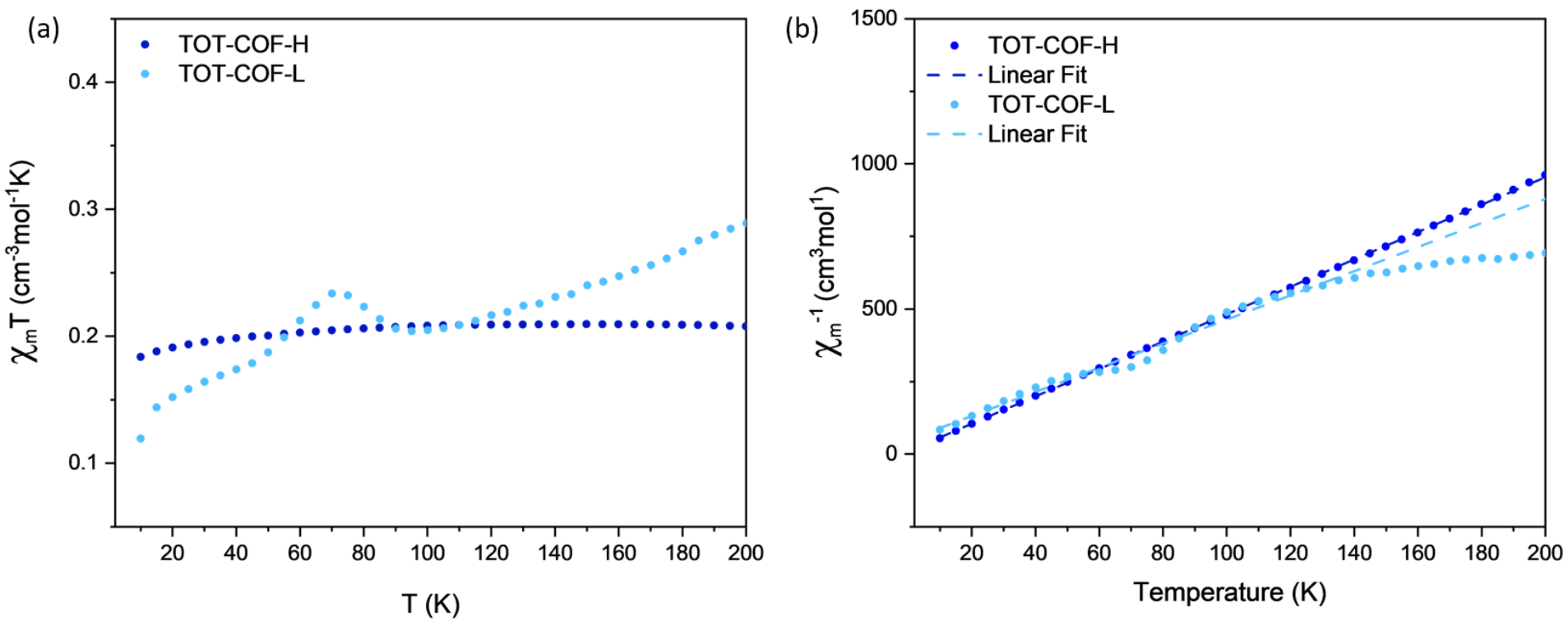

Figure S11. Magnetic susceptibility of **TOT-COF-H** and **TOT-COF-L** as a function of temperature in the 10-200 K range. (a) $\chi_m T$ versus T at H = 4000 Oe. (b) χ_m^{-1} versus T and Curie-Wiess linear fit.

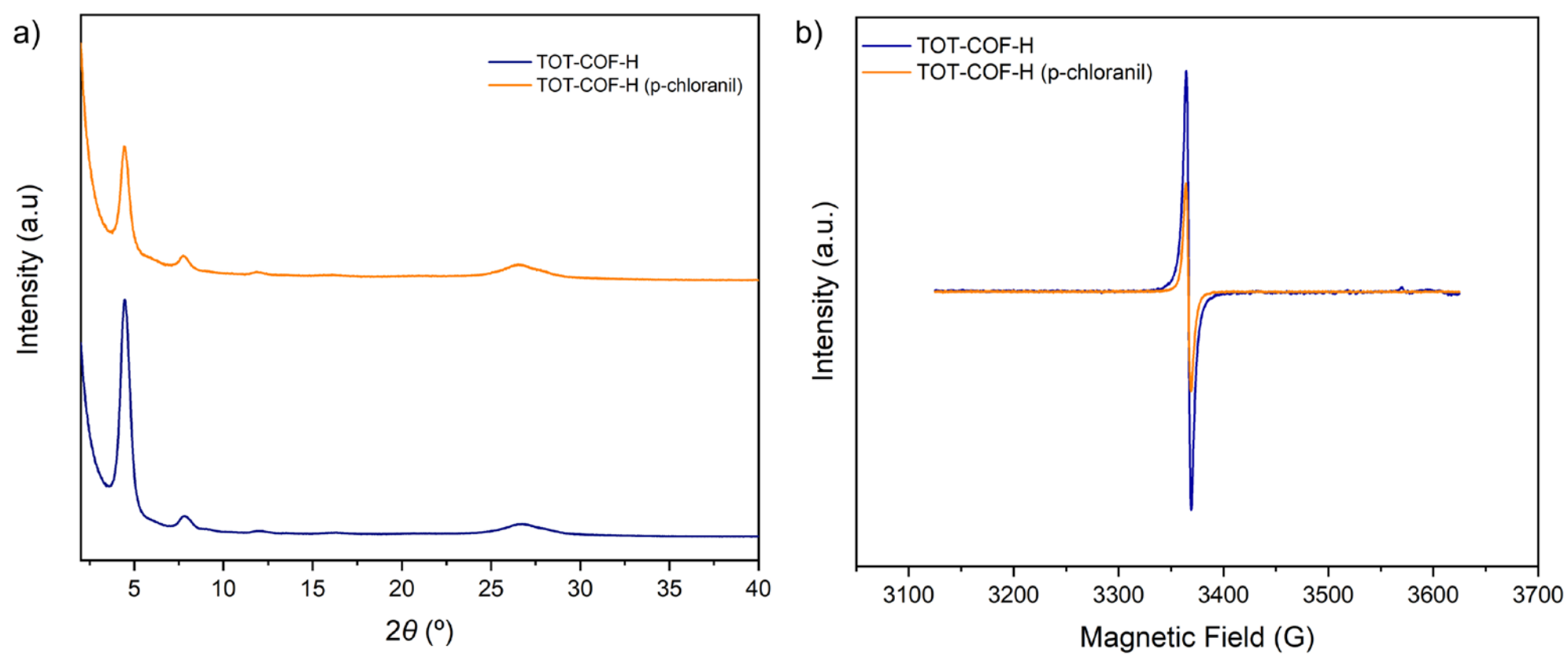

Figure S12. (a) PXRD patterns and (b) EPR spectra of TOT-COF-H before (blue) and after (orange) treatment with TBAOH in THF and oxidation with *p*-chloranil.

5. Cyclic voltammetry

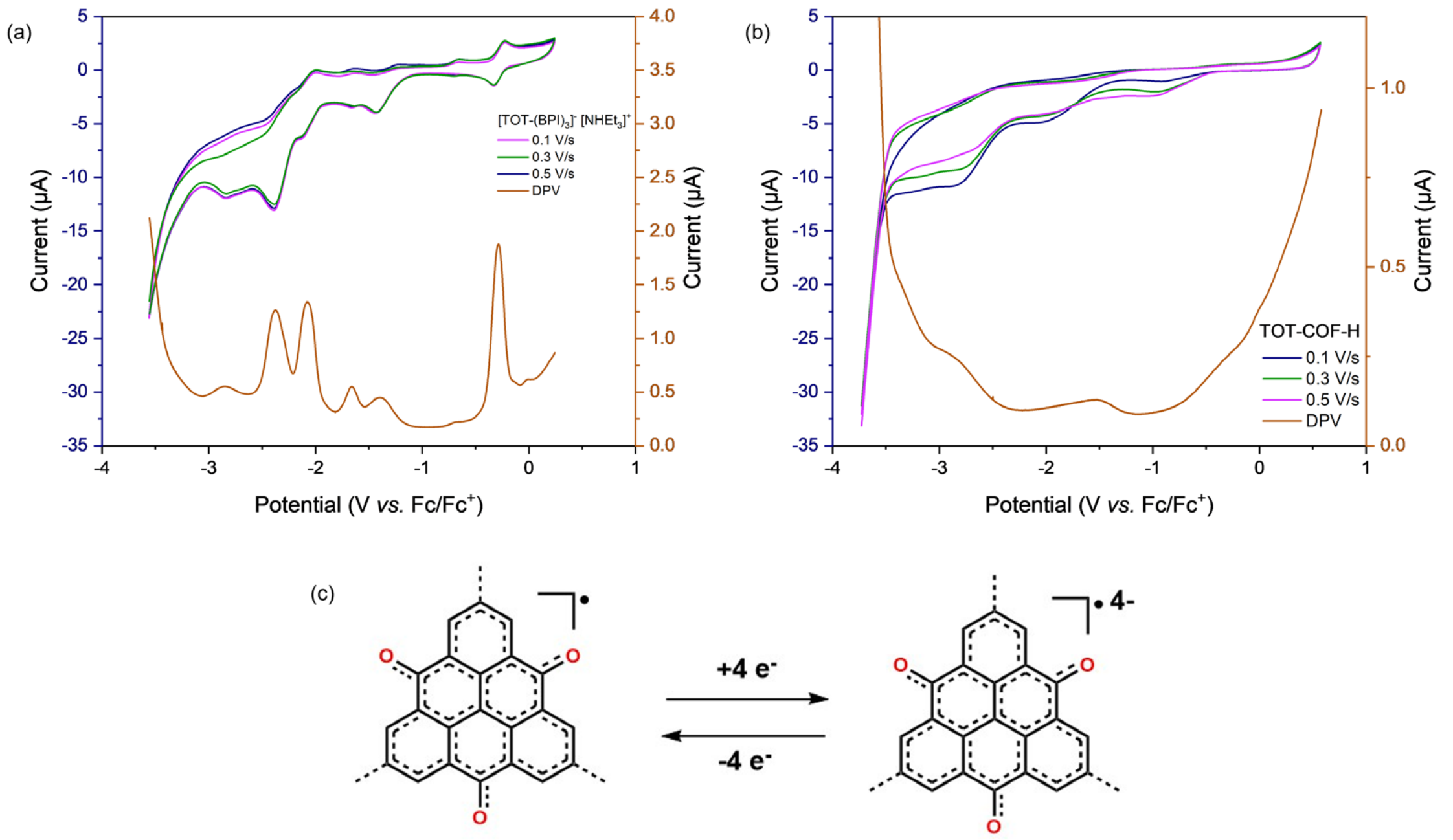

Figure S13. Cyclic voltammetry (CV) and differential pulse voltammetry (DPV) of (a) **$[TOT(BPI)_3]^-[NHEt_3]^+$** in solution and (b) **TOT-COF-H** in the solid state. 0.1 M Bu_4NClO_4 in THF was used as the electrolyte and CV was measured at different scan rates and at room temperature. Ferrocene (Fc) was used as the internal standard. (c) Consecutive one-electron redox processes of TOT radical.

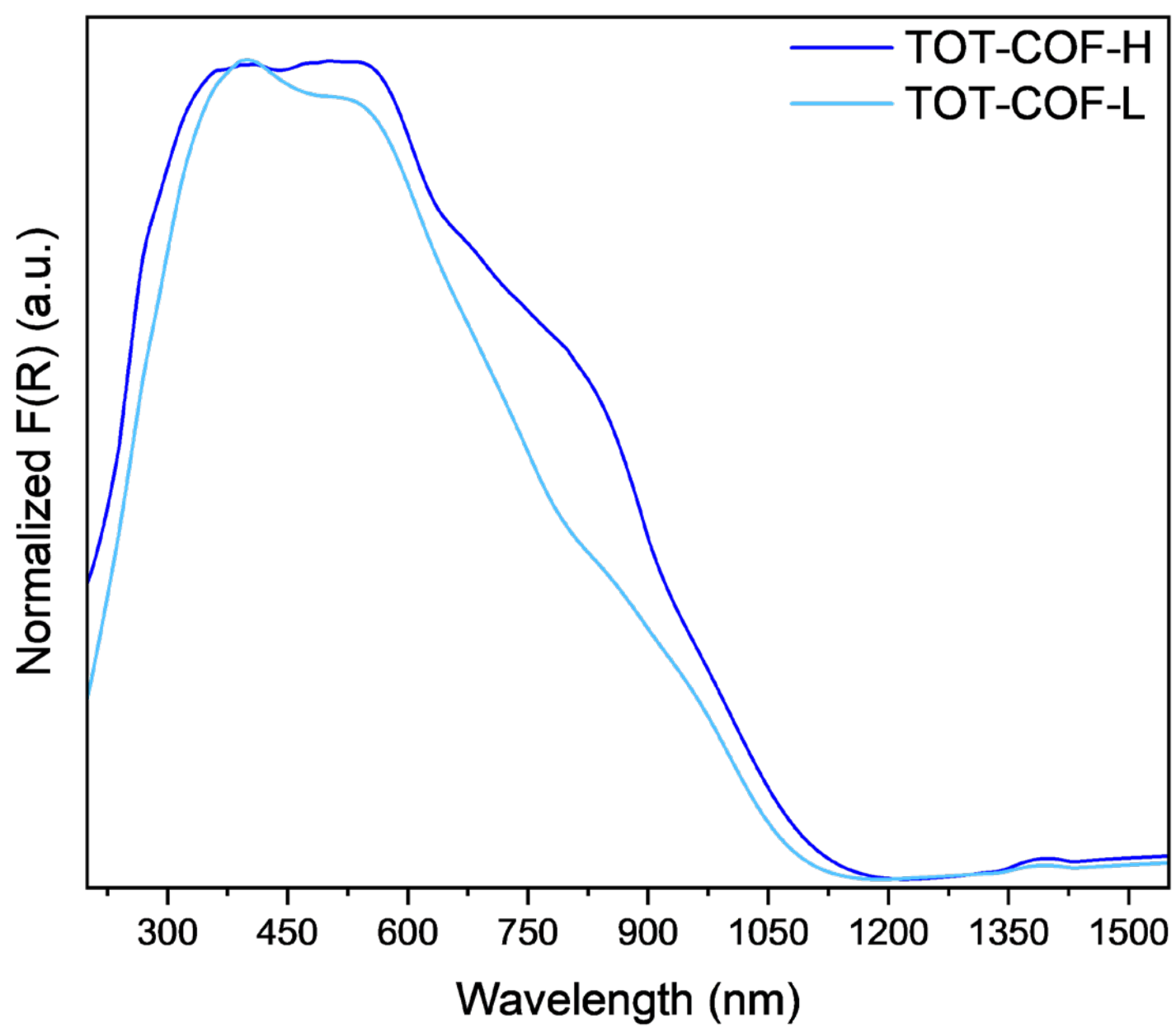

Figure S14. Normalized Kubelka-Munk-transformed spectra of **TOT-COF-H** and **TOT-COF-L** obtained from diffuse reflectance data.

6. Electrical conductivity measurements

Van der Pauw method for the determination of the electrical conductivity

Disk-shaped pellets were prepared by loading the powdered samples into a custom-built pressing die and applying a pressure of ca. 2.4 GPa using a hydraulic press. The resulting pellets had a diameter of approximately 6 mm. The pellets were placed on a Corning glass slide and mounted on the sample stage of a microprobe station (Instec HCP421V-MPS). All measurements were conducted at room temperature unless otherwise noted. For activation energy determination, the temperature dependence of the resistivity was measured over the range of 230–310 K and the activation energy extracted from the slope of the linear fit to the Arrhenius plot ($ln\rho$ vs $1/T$) as shown in Figure 3c.

Four symmetric electrical contacts were applied at the edge of each pellet using silver paste (DuPont:CP4929N-100). Electrical contacts were established by gently contacting the top surface of the silver paste contacts with gold-plated tungsten probes (see Figure S10 for the van der Pauw geometry, Table S2 and Ref. 24 for measurement definitions). The probes were connected to a Keithley 4200A-SCS Parameter Analyzer through triaxial cables. Current-Voltage (I-V) characteristics were recorded by applying a bias current and recording the resulting voltage; resistance values were extracted from the slope of the linear fit. Measurements were performed in four different configurations (Table S2), to determine the average resistance and the geometrical correction factor (*f*), which was calculated according to the following expression:

$$\frac{Q-1}{Q+1} = \frac{f}{ln2} cosh^{-1}[\frac{e^{(\frac{ln2}{f})}}{2}]$$

where $Q=R_B/R_A$ is the resistance ratio (see Table S2 for definitions).

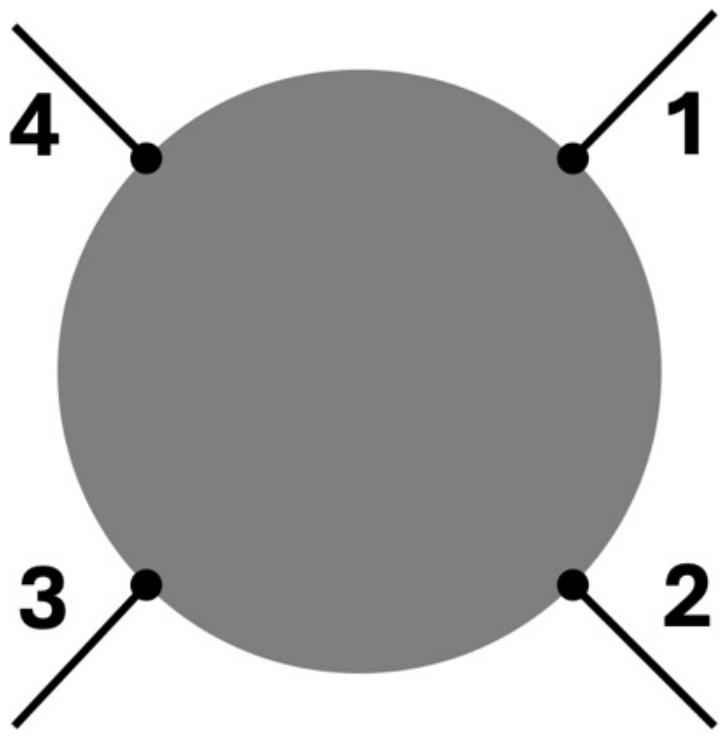

Figure S15. Schematic of the van der Pauw contact arrangement for a disk-shaped sample.

Table S3. Van der Pauw measurement definitions.

Measurement	R_1	R_3	R_2	R_4
Current sourced via probes #	1-4	2-3	1-2	4-3
Voltage measured via probes #	2-3	1-4	4-3	1-2
Resistance, $R_{A,B}$	$R_A = \frac{R_1 + R_3}{2}$		$R_B = \frac{R_2 + R_4}{2}$	

The measured I-V curves for **TOT-COF-H** and **TOT-COF-L** are shown in Figure 3 of the main manuscript. Using the procedure described above, the electrical conductivity (σ) was determined according to:

$$\sigma = \frac{ln2}{\pi t}\frac{1}{R_{ave} * f}$$

where $R_{ave} = \frac{R_A + R_B}{2}$ is the average resistance and t is the sample thickness. A summary of the obtained values is provided in Table S3.

Table S4. Electrical conductivity values obtained from four-probe van der Pauw measurements of pressed pellets (Figure 3 of the main manuscript).

**Sample**	***t*** **(mm)**	R_{ave} **(Ω)**	**Q**	***f***	***σ*** **(S/cm)**
TOT-COF-H	0.52	3.71×10^4	2.53	0.932	1.21×10^{-4}
TOT-COF-L	0.45	5.55×10^5	1.03	0.999	8.85×10^{-6}

The reproducibility of the results was assessed by performing additional electrical conductivity measurements on two different pellets of the studied materials. The resulting current–voltage (I–V) characteristics are shown in Figure S16. The asymmetry observed for the TOT-COF-H sample (Figure S16a) arises from the slightly asymmetric shape of the pellet and is taken into account in the electrical conductivity calculations using the van der Pauw method through the geometrical correction factor, (f), as described above. A summary of the obtained electrical conductivity values is provided in Table S5.

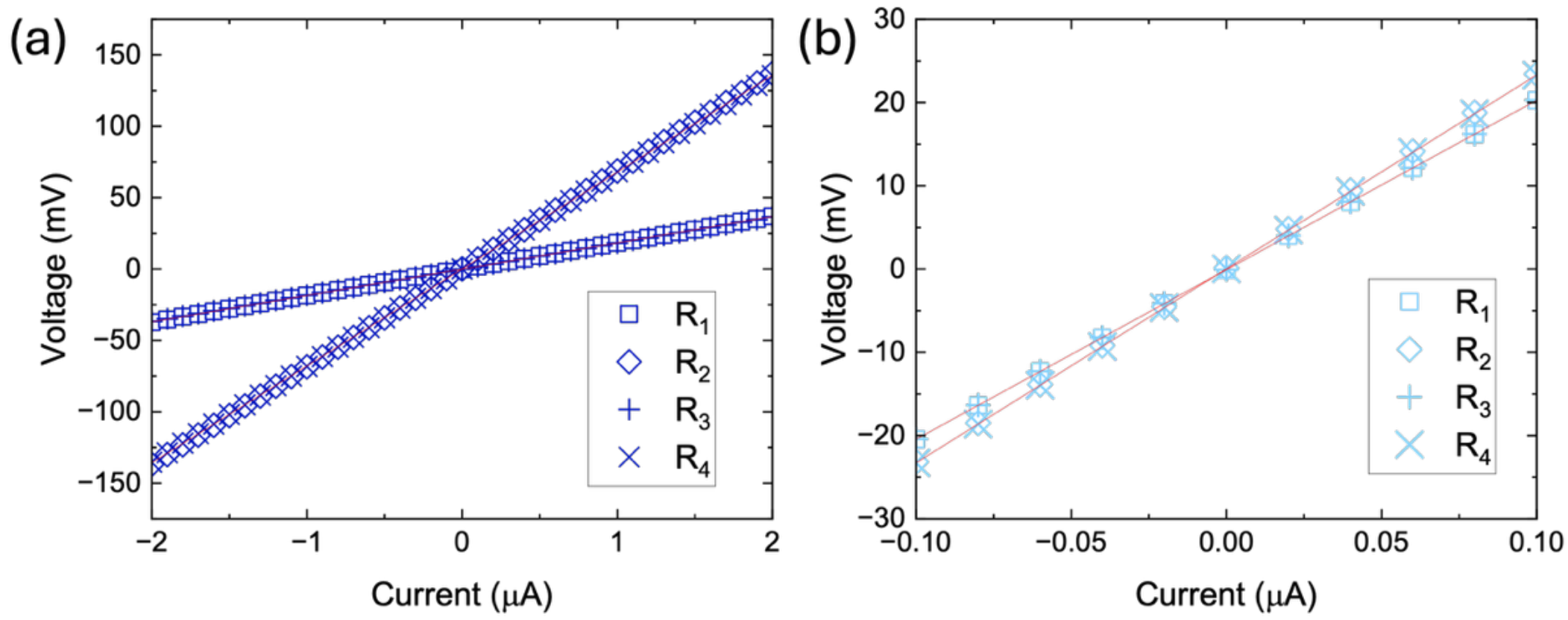

Figure S16. Current-voltage (I-V) characteristics of pressed pellets of (a) TOT-COF-H, and (b) TOT-COF-L measured using the van der Pauw method (red lines show linear fits).

Table S5. Electrical conductivity values obtained from four-probe van der Pauw measurements of pressed pellets (Fig. S16).

**Sample**	***t*** **(mm)**	R_{ave} **(Ω)**	**Q**	***f***	***σ*** **(S/cm)**
TOT-COF-H	0.52	4.33×10^4	3.71	0.873	1.12×10^{-4}
TOT-COF-L	0.50	2.18×10^5	1.14	0.999	2.01×10^{-5}

Seebeck coefficient measurements

Within semiclassical transport theory, the Seebeck coefficient reflects the energy dependence of the electronic conductivity around the Fermi level and is therefore directly related to the carrier concentration and details of the electronic structure.[4] Consequently, variations in the density of states, carrier type, or scattering mechanisms that influence charge transport are also reflected in the Seebeck coefficient. However, in contrast to electrical conductivity measurements, Seebeck measurements are performed under open-circuit conditions, with no net electrical current flowing through the sample. As a result, they are significantly less affected by intergrain boundary resistance and other microstructural effects that can strongly influence charge transport under current flow. Accordingly, the measured Seebeck coefficient more closely reflects the intrinsic transport properties of the material, with reduced contributions from intergrain boundary scattering compared to conventional electrical conductivity measurements.

In this work, the Seebeck coefficient was measured at room temperature using the same pressed pellets (≈ 0.5 mm thick) employed for electrical conductivity measurements. Each pellet was positioned between two Cu blocks, as shown in Figure S11. A thermal gradient was generated by passing a current trough an electrical resistance in one of the blocks and was accurately determined using a differential chromel/constantan thermocouple.

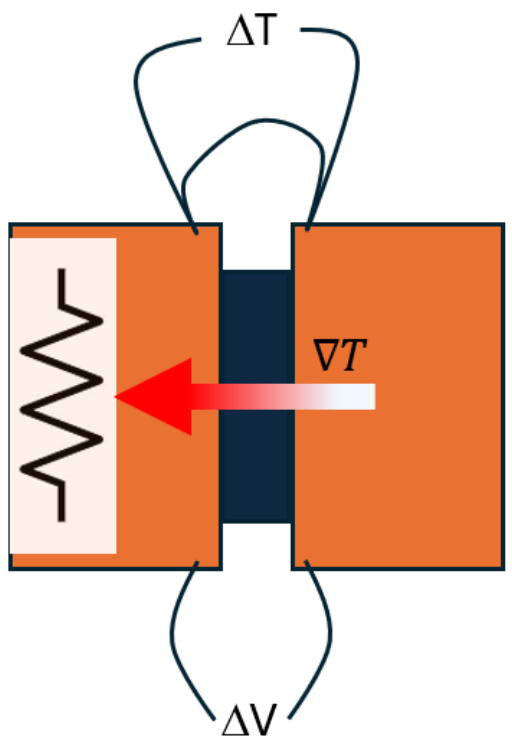

Figure S17. Schematic drawing of the Seebeck experimental setup.

The thermoelectric voltage was measured for each temperature gradient, and the Seebeck coefficient (S) was determined from the slope of the ∂V/∂ΔT curve for each sample. The system was calibrated using dense pellets of $La_{1-x}Ca_xMnO_3$ (x = 0.2 and 0.5) with known Seebeck coefficient.

Differences in absolute voltage values are not physically meaningful and mainly reflect offset voltages arising from the contacts between the pellet and the Cu blocks. Therefore, only the slope of the V vs ΔT plots, corresponding to S = ΔV/ΔT, was used to determine the Seebeck coefficient. Measurements were repeated to ensure reproducibility, and the reported uncertainties represent the statistical variance obtained from independent experiments.

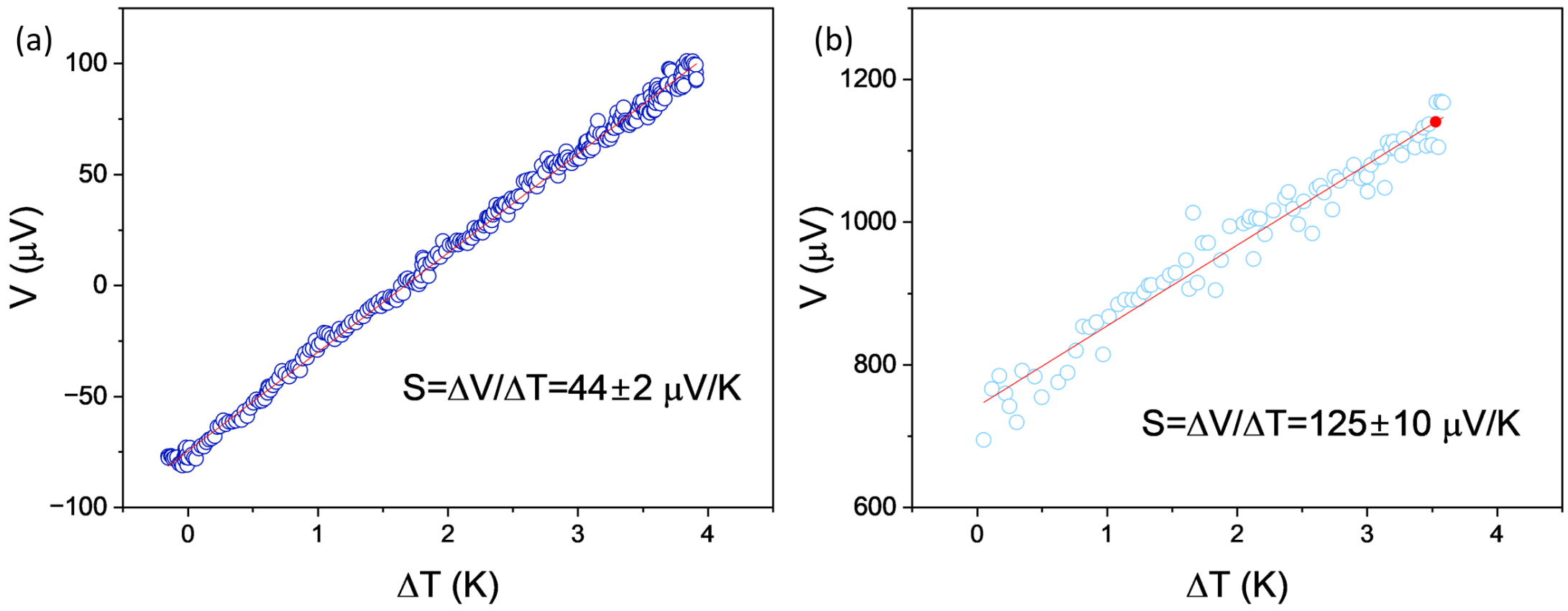

Figure S18. Seebeck coefficients of (a) **TOT-COF-H** and (b) **TOT-COF-L**. The values were determined from linear fits of the thermoelectric voltage as a function of the applied temperature gradient.

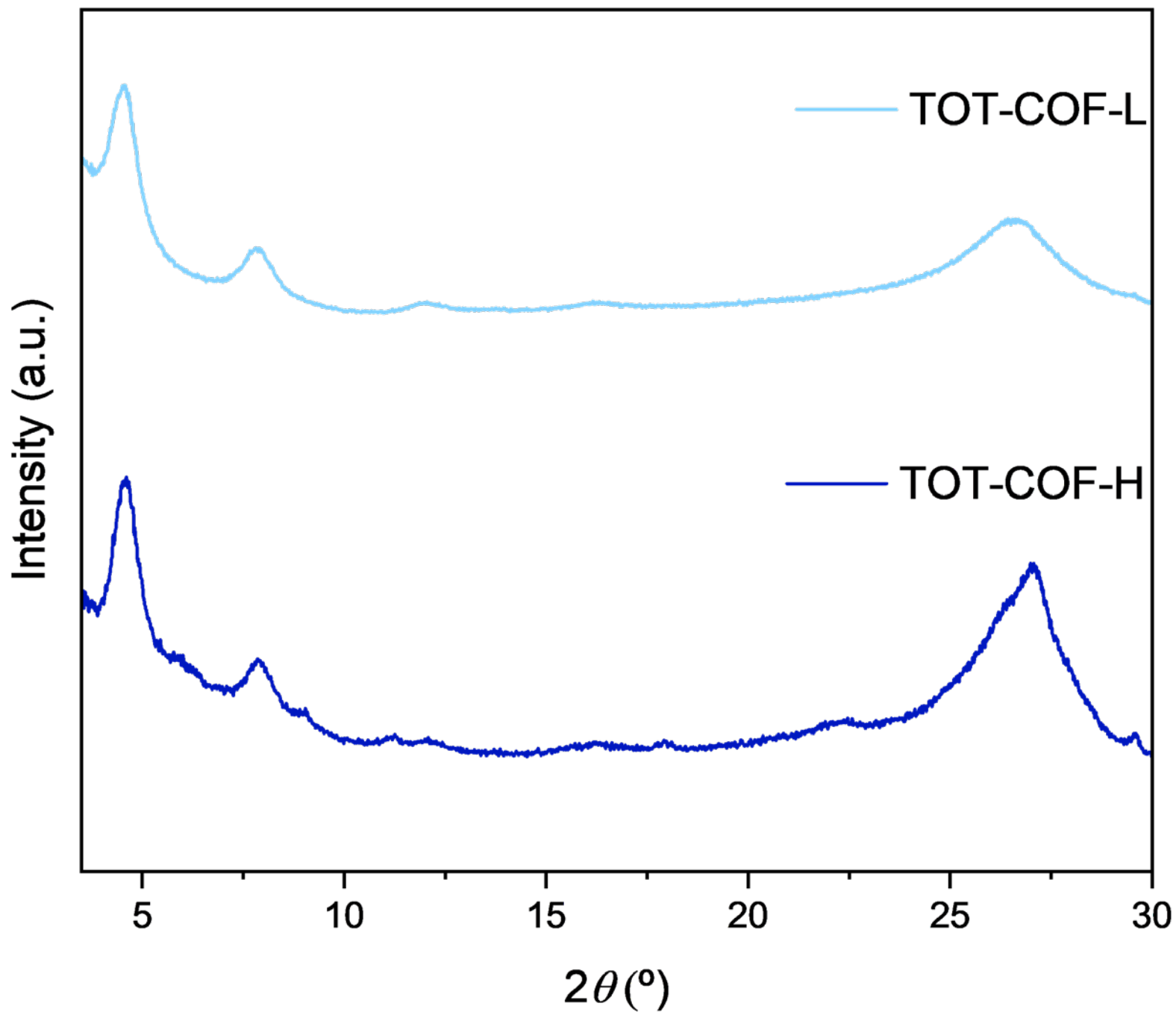

Figure S19. Experimental powder X-ray diffraction (PXRD) patterns of the pressed pellets of **TOT-COF-H** and **TOT-COF-L** after electrical conductivity measurements.

Table S6. Summary of electronic conductivity and bandgap values for some reported representative (non-doped) semiconducting 2D and 3D COFs. K–M: Kubelka–Munk transformation (from diffuse reflectance); DFT: density functional theory calculations; –: not reported.

Material	Conductivity ($S\cdot cm^{-1}$) *(pristine COFs)*	Band gap (eV)		Year (Ref.)
	Four-probes *Pellet*	Value	Method	
2D TT-COF	3.0×10^{-7}	1.22	K-M	2025 [5]
2D TTF-Ph-COF	2.66×10^{-8}	1.79	K-M	2024 [6]
2D TTF-$(Ph)_2$-COF	1.95×10^{-8}	1.84	K-M	
2D TTF-BT-COF	3.43×10^{-8}	1.82	K-M	
2D COF-DC-8	2.51×10^{-5}	--		2019 [7]
2D MC-COF-1	8×10^{-10}	1.4	K-M	2023 [8]
3D BUCT-COF-4	5.7×10^{-6}	1.85	K-M	2021 [9]
2D TFPPy-ICTO-COF	5.6×10^{-3}	0.40	DFT	2022 [10]
2D TFPPer-ICTO-COF	2.3×10^{-3}	0.55	DFT	
	Two-probes *Pellet*			
2D c-HBC-COF	$< 10^{-10}$	2.18	K-M	2022 [11]
2D TANG-COF	5.0×10^{-12}	~1.2	DFT	2020 [12]
2D TAPP−TFPP−COF	1.1×10^{-10}	--		2019 [13]
2D POR-COF	4.6×10^{-11}	--		2016 [14]
2D TAPPy-NDI	$< 10^{-9}$	1.1	DFT	2022 [15]
2D TAPB-NDI	$< 10^{-9}$	1.9	K-M	
2D TF-TA-COF	1.0×10^{-11}	1.34	K-M	2024 [16]
2D COF20-Pyr	7.6×10^{-9}	1.81	K-M	2024 [17]
2D DPP-TPPCOF	$\sim 10^{-11}$	1.63	K-M	2024 [18]
2D DPP-TBBCOF	$\sim 10^{-11}$	1.64	K-M	
1-S	3.73×10^{-10}	--		2015 [19]
1-Se	8.37×10^{-9}	--		
1-Te	1.31×10^{-7}	--		

7. Computational details

The electronic properties of TOT-COF were first investigated within density functional theory (DFT) using the Quantum ESPRESSO package (v7.5).[20] The PBE exchange-correlation functional was employed together with scalar-relativistic PAW pseudopotentials and Grimme's D3 dispersion correction with Becke–Johnson damping.[21] The TOT-COF structure was optimized both as a freestanding monolayer and as a bulk crystalline phase with a pdAA stacking arrangement. A kinetic energy cutoff of 40 Ry and charge density and potential cutoffs of 200 Ry were used, together with 3 × 3 × 3 and 3 × 3 × 1 *k*-point meshes for the bulk and monolayer calculations, respectively.

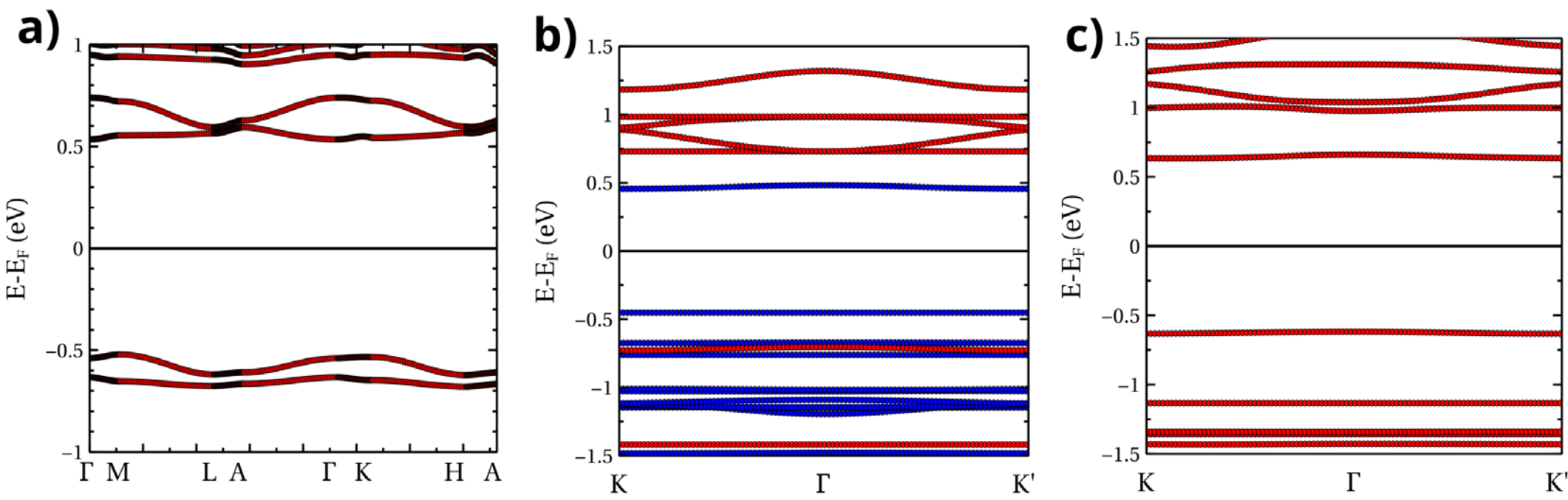

Figure S20. First principles band structures of a) non-magnetic bulk, b) magnetic monolayer, and c) non-magnetic monolayer.

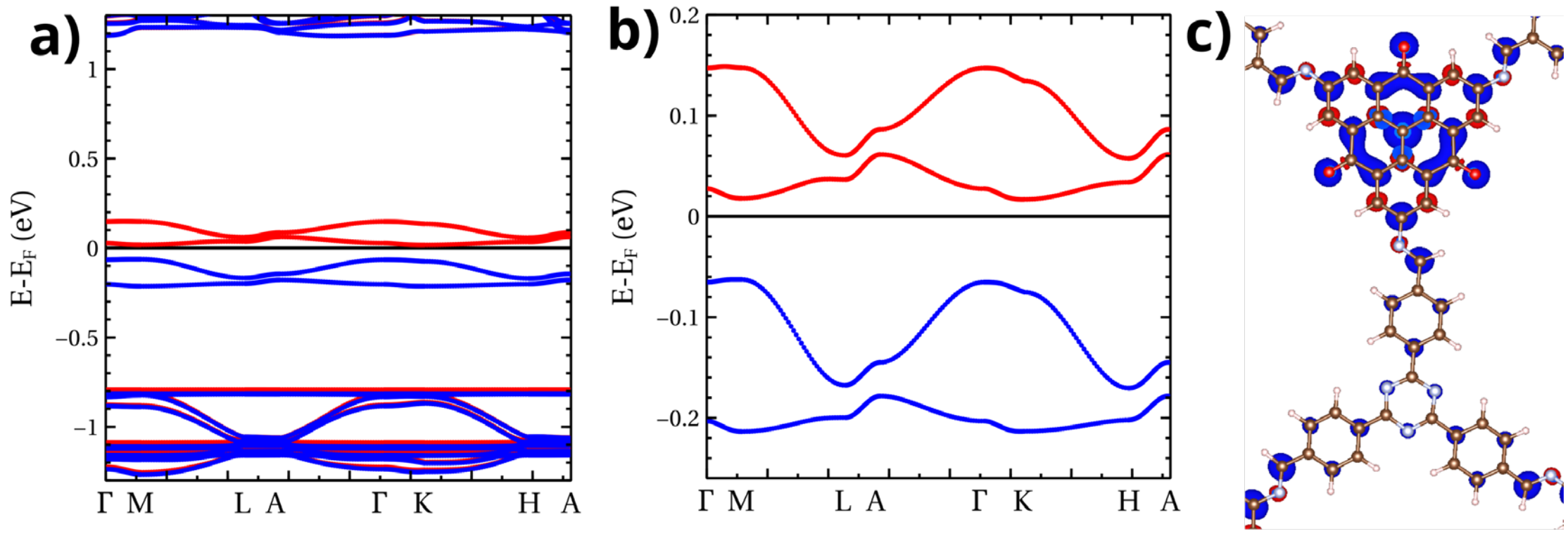

Figure S21. a) Calculated ferromagnetic band structure of bulk TOT-COF with pdAA stacking. b) Magnified view around the Fermi level, highlighting the similarity between the highest occupied and lowest unoccupied spin-up and spin-down bands. c) Spin density of monolayer TOT-COF, showing strong localization on the triangulene moiety. Red and blue denote opposite spin polarizations, consistent with the band representation.

In addition, hybrid DFT calculations were performed with the QuantumATK U-2025.06 simulation package[22] with the PBE0 exchange-correlation functional augmented by Grimme's D3 empirical dispersion correction.[21] PseudoDojo norm-conserving pseudopotentials[23], medium numerical basis sets for all atoms and a 1 × 1 × 2 *k*-point mesh were used.

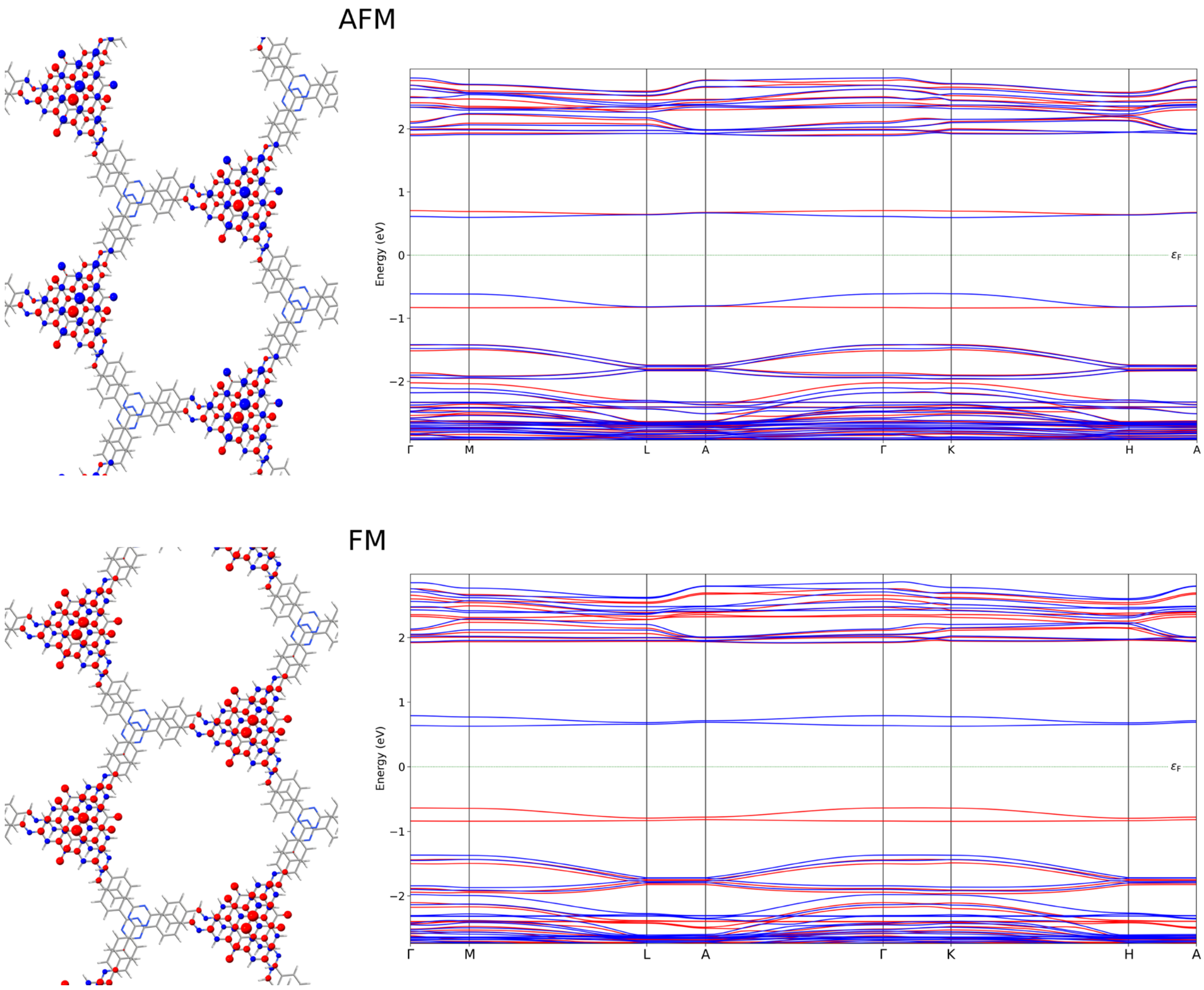

Figure S22. Computed antiferromagnetic and ferromagnetic spin densities and band structures of bulk TOT-COF with pdAA stacking, obtained using the PBE0 functional. Red and blue denote spin-up and spin-down densities, respectively.

8. References

1. Osterrieth, J. W. M.; Rampersad, J.; Madden, D.; Rampal, N.; Skoric, L.; Connolly, B.; Allendorf, M. D.; Stavila, V.; Snider, J. L.; Ameloot, R.; Marreiros, J.; Ania, C.; Azevedo, D.; Vilarrasa-Garcia, E.; Santos, B. F.; Bu, X.; Chang, Z.; Bunzen, H.; Champness, N. R.; Griffin, S. L.; Chen, B.; Lin, R. B.; Coasne, B.; Cohen, S.; Moreton, J. C.; Colón, Y. J.; Chen, L.; Clowes, R.; Coudert, F. X.; Cui, Y.; Hou, B.; D'Alessandro, D. M.; Doheny, P. W.; Dincă, M.; Sun, C.; Doonan, C.; Huxley, M. T.; Evans, J. D.; Falcaro, P.; Ricco, R.; Farha, O.; Idrees, K. B.; Islamoglu, T.; Feng, P.; Yang, H.; Forgan, R. S.; Bara, D.; Furukawa, S.; Sanchez, E.; Gascon, J.; Telalović, S.; Ghosh, S. K.; Mukherjee, S.; Hill, M. R.; Sadiq, M. M.; Horcajada, P.; Salcedo-Abraira, P.; Kaneko, K.; Kukobat, R.; Kenvin, J.; Keskin, S.; Kitagawa, S.; Otake, K.; Lively, R. P.; DeWitt, S. J. A.; Llewellyn, P.; Lotsch, B. V.; Emmerling, S. T.; Pütz, A. M.; Martí-Gastaldo, C.; Padial, N. M.; García-Martínez, J.; Linares, N.; Maspoch, D.; Suárez del Pino, J. A.; Moghadam, P.; Oktavian, R.; Morris, R. E.; Wheatley, P. S.; Navarro, J.; Petit, C.; Danaci, D.; Rosseinsky, M. J.; Katsoulidis, A. P.; Schröder, M.; Han, X.; Yang, S.; Serre, C.; Mouchaham, G.; Sholl, D. S.; Thyagarajan, R.; Siderius, D. S.; Snurr, R. Q.; Goncalves, R. B.; Telfer, S.; Lee, S. J.; Ting, V. P.; Rowlandson, J. L.; Uemura, T.; Iiyuka, T.; van der Veen, M. A.; Rega, D.; Van Speybroeck, V.; Rogge, S. M. J.; Lamaire, A.; Walton, K. S.; Bingel, L. W.; Wuttke, S.; Andreo, J.; Yaghi, O.; Zhang, B.; Yavuz, C. T.; Nguyen, T. S.; Zamora, F.; Montoro, C.; Zhou, H.; Kirchon, A.; Fairen-Jimenez, D. How Reproducible are Surface Areas Calculated from the BET Equation? *Adv. Mater*. **2022**, *34*, 2201502.

2. Murata, T.; Yokoyama, M.; Ueda, A.; Kanzaki, Y.; Shiomi, D.; Sato, K.; Takui, T.; Morita, Y. Synthesis of Trioxotriangulene Stable Neutral π-Radicals Having Alkyl Substituents Groups, and Their Effects on Electronic-spin and π -Stacking Structures. *Chem. Lett.* **2020**, *49*, 95-98.

3. Charles P. Poole, “Electron Spin Resonance. A comprehensive treatise on experimental techniques” *Dover Publications Inc.*, New York **1996**.

4. J. M. Ziman, “Electrons and Phonons: The Theory of Transport Phenomena in Solids”, *Oxford Univ. Press* **2021**.

5. Kang, C. W.; Hamzehpoor, E.; Ghamari, P.; Tao, Y.; Hossein Gohari, M.; Liu, C.; Zhang, Z.; Khaliullin, R. Z.; Perepichka, D.F. Structural Control of Band Gap and Polaron Delocalization in 2D Azatriangulene Covalent Organic Frameworks. *J. Am. Chem. Soc.* **2025**, *147*, 36320−36328.

6. Valente, G.; Dantas, R.; Ferreira, P.; Grieco, R.; Patil, N.; Guillem-Navajas, A.; Rodrí guez-San Miguel, D.; Zamora, F.; Guntermann, R.; Bein, T.; Rocha, J.; Braga, M. H.; Strutyn ski, K.; Melle-Franco, M.; Marcilla, R.; Souto, M. Tetrathiafulvalene-Based Covalent Organic Frameworks as High-Voltage Organic Cathodes for Lithium Batteries. *J. Mater. Chem. A* **2024**, *12*, 24156

7. Meng, Z.; Stolz, M. R.; Mirica, A. K. Two-Dimensional Chemiresistive Covalent Organic Framework with High Intrinsic Conductivity. *J. Am. Chem. Soc* **2019**, *141*, 11929-11937.

8. Huang, S.; Choi, J. Y.; Xu, Q.; Jin, Y.; Park, J.; Zhang, W. Carbazolylene-Ethynylene Macrocycle-Based Conductive Covalent Organic Frameworks. *Angew. Chem. Int. Ed.* **2023**, *62*, e202303538.

9. Wang, S.; Li, X.-X.; Da, L.; Wang, Y.; Xiang, Z.; Wang, W.; Zhang, Y.-B.; Cao, D. A Three-Dimensional sp2 Carbon-Conjugated Covalent Organic Framework. *J. Am. Chem. Soc.* **2021**, *143*, 15562–15566.

10. Xu, X.; Zhang, S.; Xu, K.; Chen, X.; Fan, X.; Huang, N. Janus Dione-Based Conjugated Covalent Organic Frameworks with High Conductivity as Superior Cathode Materials. *J. Am. Chem. Soc.* **2023**, *145*, 1022-1030.

11. Xing, G.; Zheng, W.; Gao, L.; Zhang, T.; Wu, X.; Fu, S.; Song, X.; Zhao, Z.; Osella, S.; Martí nez-Abadí a, M.; Wang, H. I.; Cai, J.; Mateo-Alonso, A.; Chen, L. Nonplanar Rhombus and Kagome 2D Covalent Organic Frameworks from Distorted Aromatics for Electrical Conduction. *J. Am. Chem. Soc.* **2022**, *144*, 5042–5050.

12. Lakshmi, V.; Liu, C.-H.; Rao, M. R.; Chen, Y.; Fang, Y.; Dadvand, A.; Hamzehpoor, E.; Sakai-Otsuka, Y.; Stein, R. S.; Perepichka, D. F. A Two-Dimensional Poly(azatriangulene) Covalent Organic Framework with Semiconducting and Paramagnetic States. *J. Am. Chem. Soc.* **2020**, *142*, 2155–2160.

13. Xu, X.; Wang, S.; Yue, Y.; Huang, N. Semiconductive Porphyrin-Based Covalent Organic Frameworks for Sensitive Near-Infrared Detection. *ACS Appl. Mater. Interfaces* **2020**, *12*, 37427–37434.

14. Nath, B.; Li, W.-H.; Huang, J.-H.; Wang, G.-E.; Fu, Z.-H.; Yao, M.-S.; G. A new azodioxy-linked porphyrin-based semiconductive covalent organic framework with I_2 doping-enhanced photoconductivity. *CrystEngComm*, 2016,**18**, 4259-4263.

15. Evans, A. M.; Collins, K. A.; Xun, S.; Allen, T. G.; Jhulki, S.; Castano, I.; Smith, H. L.; Strauss, M. J.; Oanta, A. K.; Liu, L.; Sun, L.; Reid, O. G.; Sini, G.; Puggioni, D.; Rondinelli, J. M.; Gianneschi, N. C.; Kahn, A.; Freedman, D. E.; Li, H.; Barlow, S.; Rumbles, G.; Bre das, J.-L.; Marder, S. R.; Dichtel, W. R. Controlled n-Doping of Naphthalene-Diimide-Based 2D Polymers. *Adv. Mater.* **2022**, *34*, 2101932.

16. Li, Y.; Cui, G.; Cai, X.; Yun, G.; Zhao, Y.; Jiang, L.; Cui, S.; Zhang, J.; Liu, M.; Zeng, W.; Wang, Z.; Jiang, J. A New Porphyrin-Based Covalent Organic Framework with High Iodine Capture Capacity and I-Doping Enhanced Conductivity. *Chem. Eur. J.* **2024**, *30*, e202303688.

17. Jain, C.; Kushwaha, R.; Rase, D.; Shekhar, P.; Shelke, A.; Sonwani, D.; Ajithkumar, T. G.; Vinod, C. P.; Vaidhyanathan, R. Tailoring COFs: Transforming Nonconducting 2D Layered COF into a Conducting Quasi-3D Architecture via Interlayer Knitting with Polypyrrole. *J. Am. Chem. Soc.* **2024**, *146*, 487–499.

18. Luo, L.; Li, C.; Wang, Y.; Chen, P.; Zhou, Z.; Chen, T.; Wu, K.; Ding, S.-Y.; Tan, L.; Wang, J.; Shao, X.; Liu, Z. Multi-Functional 2D Covalent Organic Frameworks with Diketopyrrolopyrrole as Electron Acceptor. *Small* **2024**, *20*, 2402993.

19. Duhović, S.; Dincă, M. Synthesis and Electrical Properties of Covalent Organic Frameworks with Heavy Chalcogens. *Chem. Mater*. **2015**, *27*, 5487-5490.

20. P. Giannozzi et al., *J. Phys.: Condens. Matter* **2009**, *21*, 395502.

21. S. Grimme, S. Ehrlich, L. Goerigk, *J. Comput. Chem.* **2011**, *32*, 1456–1465.

22. S. Smidstrup et al., *J. Phys.: Condens. Matter* **2020**, 32, 015901**.**

23. M.J. van Setten et al., *Comp. Phys. Comm.* **2018**, 226, 39-54.

24. Van der Pauw, L.J. A method of measuring specific resistivity and Hall effect of discs of arbitrary shape. *Philips Research Reports* **1958**, 13, 1-9
